# Functional dissection of human cardiac enhancers and noncoding de novo variants in congenital heart disease

**DOI:** 10.1038/s41588-024-01669-y

**Published:** 2024-02-20

**Authors:** Feng Xiao, Xiaoran Zhang, Sarah U. Morton, Seong Won Kim, Youfei Fan, Joshua M. Gorham, Huan Zhang, Paul J. Berkson, Neil Mazumdar, Yangpo Cao, Jian Chen, Jacob Hagen, Xujie Liu, Pingzhu Zhou, Felix Richter, Yufeng Shen, Tarsha Ward, Bruce D. Gelb, Jonathan G. Seidman, Christine E. Seidman, William T. Pu

**Affiliations:** 1 Department of Cardiology, Boston Children’s Hospital, Boston, MA, USA.; 2 Department of Pediatrics, Harvard Medical School, Boston, MA, USA.; 3 Division of Newborn Medicine, Boston Children’s Hospital, Boston, MA, USA; 4 Department of Genetics, Harvard Medical School, Boston, MA, USA.; 5 Department of Pediatrics, Shandong Provincial Hospital Affiliated to Shandong First Medical University, Jinan, China.; 6 Department of Radiation Oncology, Dana–Farber Cancer Institute, Boston, MA, USA.; 7 Mindich Child Health and Development Institute and Department of Pediatrics, Icahn School of Medicine at Mount Sinai, New York City, NY, USA.; 8 Department of Systems Biology and Biomedical Informatics, Columbia University Medical Center, New York City, NY, USA.; 9 Department of Genetics and Genomic Sciences, Icahn School of Medicine at Mount Sinai, New York City, NY, USA.; 10 Division of Cardiology, Brigham and Women’s Hospital, Boston, MA, USA.; 11 Howard Hughes Medical Institute, Chevy Chase, MD, USA.; 12 Harvard Stem Cell Institute, Cambridge, MA, USA.; 13 Present address: Department of Pharmacology, School of Medicine, Southern University of Science and Technology, Shenzhen, China.; 14 These authors contributed equally: Feng Xiao, Xiaoran Zhang, Sarah U. Morton.

## Abstract

Rare coding mutations cause ~45% of congenital heart disease (CHD). Noncoding mutations that perturb *cis*-regulatory elements (CREs) likely contribute to the remaining cases, but their identification has been problematic. Using a lentiviral massively parallel reporter assay (lentiMPRA) in human induced pluripotent stem cell-derived cardiomyocytes (iPSC-CMs), we functionally evaluated 6,590 noncoding de novo variants (ncDNVs) prioritized from the whole-genome sequencing of 750 CHD trios. A total of 403 ncDNVs substantially affected cardiac CRE activity.

A majority increased enhancer activity, often at regions with undetectable reference sequence activity. Of ten DNVs tested by introduction into their native genomic context, four altered the expression of neighboring genes and iPSC-CM transcriptional state. To prioritize future DNVs for functional testing, we used the MPRA data to develop a regression model, EpiCard. Analysis of an independent CHD cohort by EpiCard found enrichment of DNVs. Together, we developed a scalable system to measure the effect of ncDNVs on CRE activity and deployed it to systematically assess the contribution of ncDNVs to CHD.

Congenital heart disease (CHD), the most common birth defect, affects almost 1% of all live births^1^. Whole exome sequencing of parent–offspring trios demonstrated protein-damaging, de novo variants (DNVs) that are enriched in CHD probands, especially in genes that are highly expressed in the heart during development (high heart expressed (HHE) genes)^2–4^. These and other studies demonstrated that rare coding variants account for ~45% of CHD cases.

Approximately 99% of the human genome consists of noncoding DNA^5^. To consider the potential influence of noncoding variants in CHD, the Pediatric Cardiac Genomics Consortium (PCGC) defined DNVs through analyses of whole-genome sequencing (WGS) in CHD probands and parents^6^. By prioritizing DNVs predicted to affect *cis*-regulatory elements (CREs) of genes implicated in CHD, we identified an increased burden of noncoding DNVs (ncDNVs) among patients with CHD. However, as there are ~74 ncDNVs per individual^6,7^, distinguishing likely pathogenic ncDNVs from background genetic variation remains challenging in the absence of comprehensive functional evaluation of candidate CRE regions. The relatively lower conservation of cardiac CREs^8^ and the potential for species-dependent effects of noncoding variants are additional barriers. Key tools needed to expedite the evaluation of the functional impact of ncDNVs are computational approaches to effectively prioritize variants for burden or functional testing^9–12^ and high-throughput platforms to measure the impact of ncDNVs on CRE activity in human cells^13^.

Here we investigated the contribution of ncDNVs to CHD by developing a high-throughput platform to measure CRE activity in human-induced pluripotent stem cell-derived cardiomyocytes (hiPSC-CMs). We leveraged this platform to interrogate 6,590 ncDNVs prioritized from CHD trios and identified 403 ncDNVs that substantially affected CRE activity in iPSC-CMs. We introduced ten of these ncDNVs into hiPS cells and found that four influenced adjacent gene expression and transcriptional state of iPSC-CMs. Using these data, we developed a model to predict CRE activity. This predictor outperformed previously developed methods and identified increased burden of ncDNVs in a second, independent CHD cohort. Collectively, our study advanced the evaluation of human cardiac enhancer activity and provided new insights into CHD pathogenesis.

## Results

### lentiMPRA to measure enhancer activity in hiPSC-CMs

**Extended Data Fig. 1 |:**
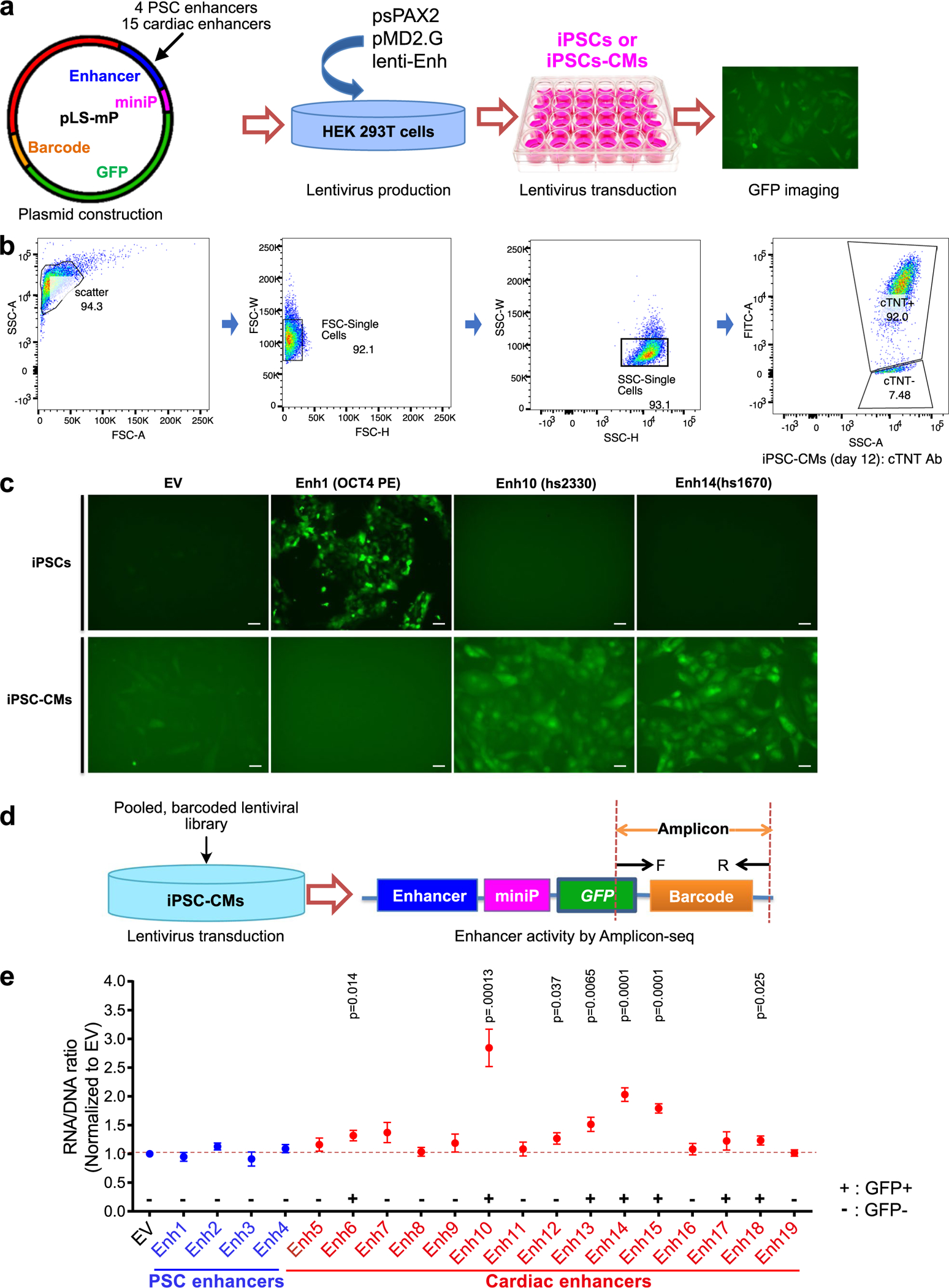
Establishment of the lentiMPRA platform to test cardiac enhancer activity in iPSC-CMs. **a**. Strategy for pilot experiment to test lentiviral reporter assay in iPSC-CMs. **b**. Flow cytometry analysis of cTNT+ iPSC-CMs at differentiation day 12. Cells were gated with SSC and FSC to exclude debris and doublets. Flow cytometry plots displayed a biomodal distribution between fluorescent and non-fluorescent cells. Gates determining the percent of fluorescent cells were drawn at the local minimum between these distributions. **c**. Activities of PSC-specific enhancer (OCT4 PE) and cardiac enhancers (VISTA enhancer browser hs2330 and hs1670) in iPSCs and iPSC-CMs. Representative images from 4 independent experiments. Scale bar, 100 μm. **d**. Strategy for pilot experiment to measure enhancer activity by Amplicon-seq. **e**. Enhancer activities of PSC enhancers (Enh1–4) and cardiac enhancers (Enh 5–19). Activity of the empty vector (EV) was set 1. Enhancer activity was normalized to EV. Data are represented as mean ± SEM of 4 independent experiments (2-sided unpaired t test).

We established a platform for high-throughput measurement of CRE activity by deploying a lentiviral massively parallel reporter assay (lentiMPRA) in human iPSC-CMs^14,15^. Lentivirus efficiently transduces iPS cells and iPSC-CMs and integrates into the genome, allowing enhancers to be assayed in a chromosomal rather than episomal context^14^. We initially piloted this platform by cloning four verified human pluripotent stem cell (PSC)-specific enhancers^16^ and 15 human cardiac enhancers validated by mouse transient transgenesis^17^ into a lentiMPRA vector containing a minimal promoter, green fluorescent protein (GFP) reporter gene, and barcodes in the 3′ UTR uniquely matched to the cloned enhancers (Extended Data Fig. 1a and Supplementary Table 1). This pilot experiment verified that PSC enhancers were active in iPS cells but not iPSC-CMs, and a subset of cardiac enhancers were active in iPSC-CMs but not iPS cells (Extended Data Fig. 1b,c). Quantitation of enhancer activity in iPSC-CMs by barcode frequency in RNA compared to genomic DNA corresponded to qualitative GFP fluorescence (Extended Data Fig. 1d,e).

**Extended Data Fig. 2 |:**
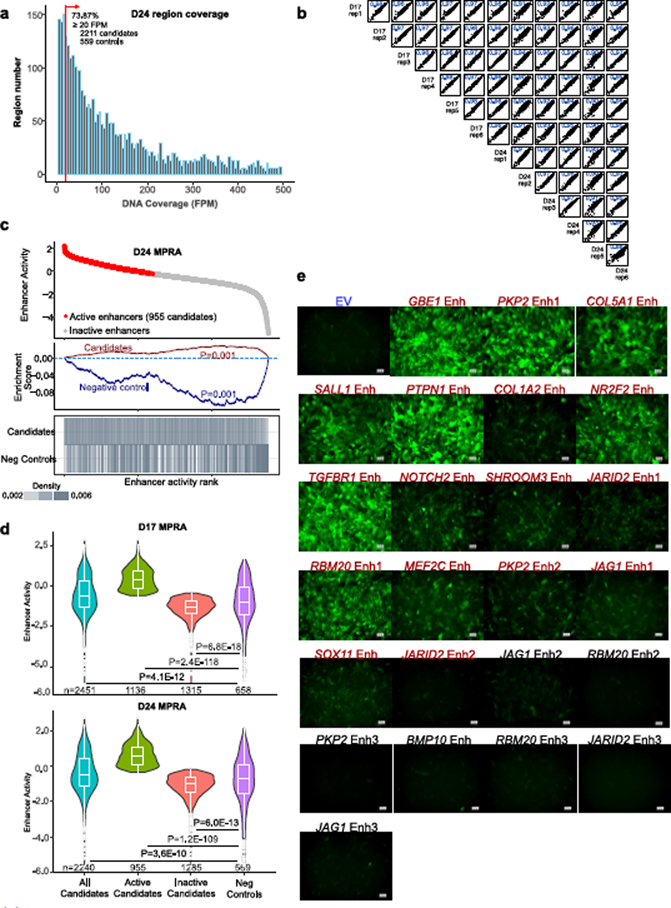
Assessment of human cardiac enhancer activity with hiPSC-CMs and lentiSTARR-seq. **a**. Minimal read coverage of designed regions in DNA replicates. Red line shows minimum coverage for inclusion in analysis (FPM ≥ 20). **b**. Pearson correlation of MPRA activity between biological replicates at D17 and D24. There was excellent correlation both within group and across time points. **c**. Summary of MPRA results. Plot at the bottom shows a vertical line for each tested region with the indicated annotation. Enrichment score indicates enrichment of a set of regions of interest toward the ends of the ranked list of all regions. Enrichment p-value was determined by 1-sided permutation test (see Methods) with Bonferroni correction. Active enhancers were those enriched in RNA compared to DNA (DESeq2 *P*_adj_ < 0.05). **d**. Violin plot with the log_2_(RNA/DNA) results of all candidates, active candidates, inactive candidates and negative controls. Kruskal-Wallis test p-values vs. neg control are shown. Center, box and whiskers indicate median, 25th and 75th percentiles and value closest to 25th percentile minus or 75th percentile plus 1.5 times the interquartile range. **e**. Twenty-four candidate cardiac enhancers of known cardiovascular disease genes with a range of MPRA enhancer activity were individually cloned into the lentiMPRA vector, in which a minimal promoter drives GFP expression. Red color indicates enhancers that were classified as active by MPRA. GFP expression was evaluated by epifluorescent imaging. Representative images from 4 independent experiments. Scale bar, 100 μm.

**Fig. 1 |:**
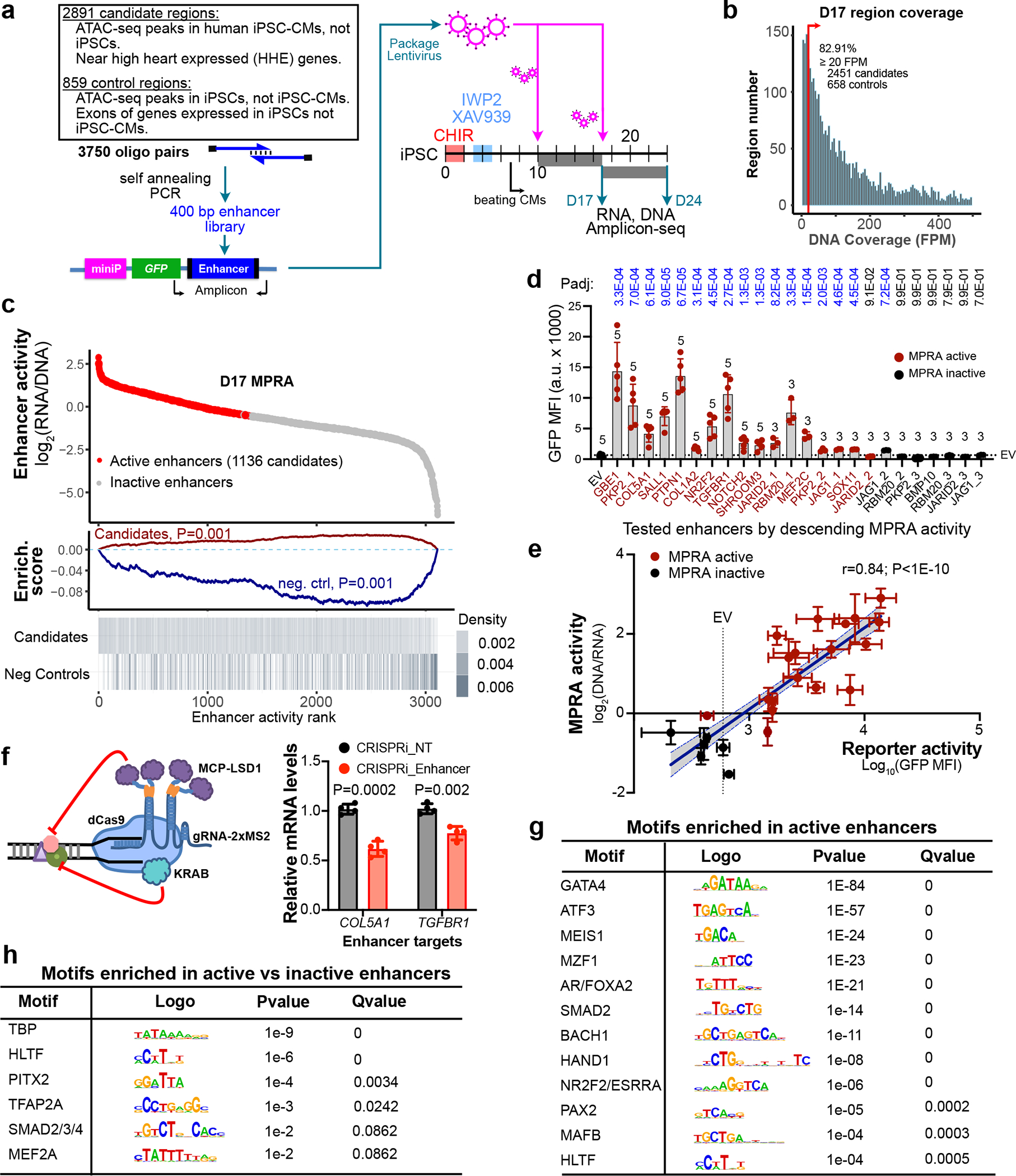
Assessment of human cardiac enhancer activity with hiPSC-CMs and lentiSTARR-seq. **a**, Experimental design of lentiSTARR-seq of candidate cardiac enhancers in iPSC-CMs. **b**, Coverage of designed regions. Red line shows minimum coverage in amplicons from genomic DNA for inclusion in analysis (FPM ≥ 20). **c**, Summary of lentiSTARR-seq results. Top plot shows the enhancer activity of each region, as a function of activity rank. Active enhancers—enhancers enriched in RNA compared to DNA (DESeq2 (ref. 37) *P*_adj_ < 0.05)—are colored red. Bottom line plot shows a vertical line, colored by count density, for each tested region with the indicated annotation. Enrichment significance was determined by one-way permutation test with Bonferroni correction (Methods). **d**,**e**, LentiSTARR-seq validation. Seventeen active and seven inactive enhancers neighboring cardiovascular disease genes were cloned individually into the lentiMPRA vector. iPSC-CMs were transduced on day 17 and assayed on day 24. **d**, GFP fluorescence of the empty vector control and enhancer-reporter lentiviruses was measured by flow cytometry. The numbers above bars show a number of independent biological replicates. Numbers at the top show one-sided *t*-test for activity above empty vector with Benjamini–Hochberg multiple testing correction. Blue indicates *P*_adj_ < 0.05. **e**, Correlation of enhancer activity measured by GFP MFI (sample sizes shown in **d**) and by MPRA (*n* = 4 biological replicates). Black line shows the best fit linear regression line and 95% confidence interval. **f**, Functional validation of two enhancers neighboring *COL5A1* and *TGFBR1* using CRISPRi with KRAB and LSD1. NT, nontargeting control gRNA. *n* = 4. Two-sided *t*-test with Bonferroni correction. **g**,**h**, Motif analysis of active candidate enhancers using genomic background (**g**) or inactive enhancers as background (**h**). The active enhancers were the union of the candidate regions active in the day 17 and day 24 experiments (*n* = 1,185). Motif enrichment *P* value was calculated by Homer^38^ using a binomial distribution and Benjamini–Hochberg correction (*Q* value). For complete motif analysis results, see Supplementary Data 1. Data are shown as mean ± s.d. MFI, mean fluorescence intensity.

To apply this platform to the high-throughput measurement of cardiac CRE activity, we reconfigured the lentiviral vector as a self-transcribing active regulatory region sequencing (lentiSTARR-seq) vector in which the enhancer is positioned in the reporter gene’s 3′ UTR and serves as its own barcode^18^ and used it to measure the enhancer activity of 2,891 candidate human cardiac enhancers and 859 negative controls (Fig. 1a). The candidate CRE sequences were located in assay for transposase-accessible chromatin using sequencing (ATAC-seq) peaks in iPSC-CMs but not iPS cells^6^, did not contain coding sequences or promoters and neighbored genes in the top quartile of heart expression^2^. The negative controls were chosen from regions accessible in iPS cells but not iPSC-CMs or from exons highly expressed in iPS cells but not iPSC-CMs (Fig. 1a and Supplementary Data 1). Test regions were created by pooled oligo synthesis of a pair of 230 nt oligonucleotides, which were extended to 400 bp by self-priming PCR (Fig. 1a). The pool of PCR amplified regions were cloned into the 3′ UTR of the lentiSTARR-seq vector^18^ (Fig. 1a). The lentiSTARR-seq library was introduced into iPSC-CMs at day 10 or 17 of differentiation, and cells were collected 7 days later. The 3′ UTR of the reporter gene containing the candidate CREs was amplified from RNA and genomic DNA and sequenced. We filtered out regions with insufficient library coverage (17.1% of regions; Fig. 1b and Extended Data Fig. 2a). Enhancer activity was calculated by its frequency in RNA compared to DNA. Activity measurements from six biological replicates were highly reproducible between replicates and time points (Pearson *r* = 0.95 ± 0.03; Extended Data Fig. 2b and Supplementary Data 1). Defining active regions as those overrepresented in RNA compared to DNA^19^ yielded 1,136 and 955 active cardiac enhancers at days 17 and 24, respectively (Fig. 1c and Extended Data Fig. 2c,d).

A recent comparison of MPRA designs suggested that the lentiSTARR-seq design only moderately correlated (Pearson *r* = 0.60) with other designs^20^. Therefore we extensively validated the lentiSTARR-seq results. We selected 24 cardiovascular disease gene-associated regions with a range of activities in the lentiSTARR-seq assay and tested them individually by cloning them into the lentiMPRA vector. In iPSC-CMs, GFP fluorescence of active regions, quantified by fluorescence-activated cell sorting (FACS), was substantially above that of empty vector in 16 out of 17 regions tested (94%), and inactive regions were comparable to or less than the empty vector in 6 out of 7 regions tested (86%; Fig. 1d, Extended Data Fig. 2e and Supplementary Table 2). Indeed, GFP fluorescence intensity and MPRA activity were strongly correlated (Fig. 1e). We targeted two validated active enhancers that neighbored *COL5A1* and *TGFBR1*, known CHD genes, using CRISPR interference^21^ (CRISPRi; Fig. 1f). Guides targeting these enhancers reduced their expression, whereas nontargeting guides did not (Fig. 1f), indicating that these enhancers are essential transcriptional activators of these genes.

To better understand the features of these active cardiac enhancers, we performed transcription factor motif analysis. Motifs enriched in active enhancers compared to genomic background included those of GATA4, SMAD2, MEIS1, HAND1 and MEF2, transcription factors important for heart development (Fig. 1g and Supplementary Data 1). Of these, MEF2 and SMAD2 were also enriched in active compared to inactive regions (Fig. 1h and Supplementary Data 1).

Collectively, these data show that lentiMPRA combined with hiPSC-CMs is an effective high-throughput platform to assess human cardiac enhancer activity.

### Analysis of human cardiac enhancers by tiling mutagenesis

**Extended Data Fig. 3 |:**
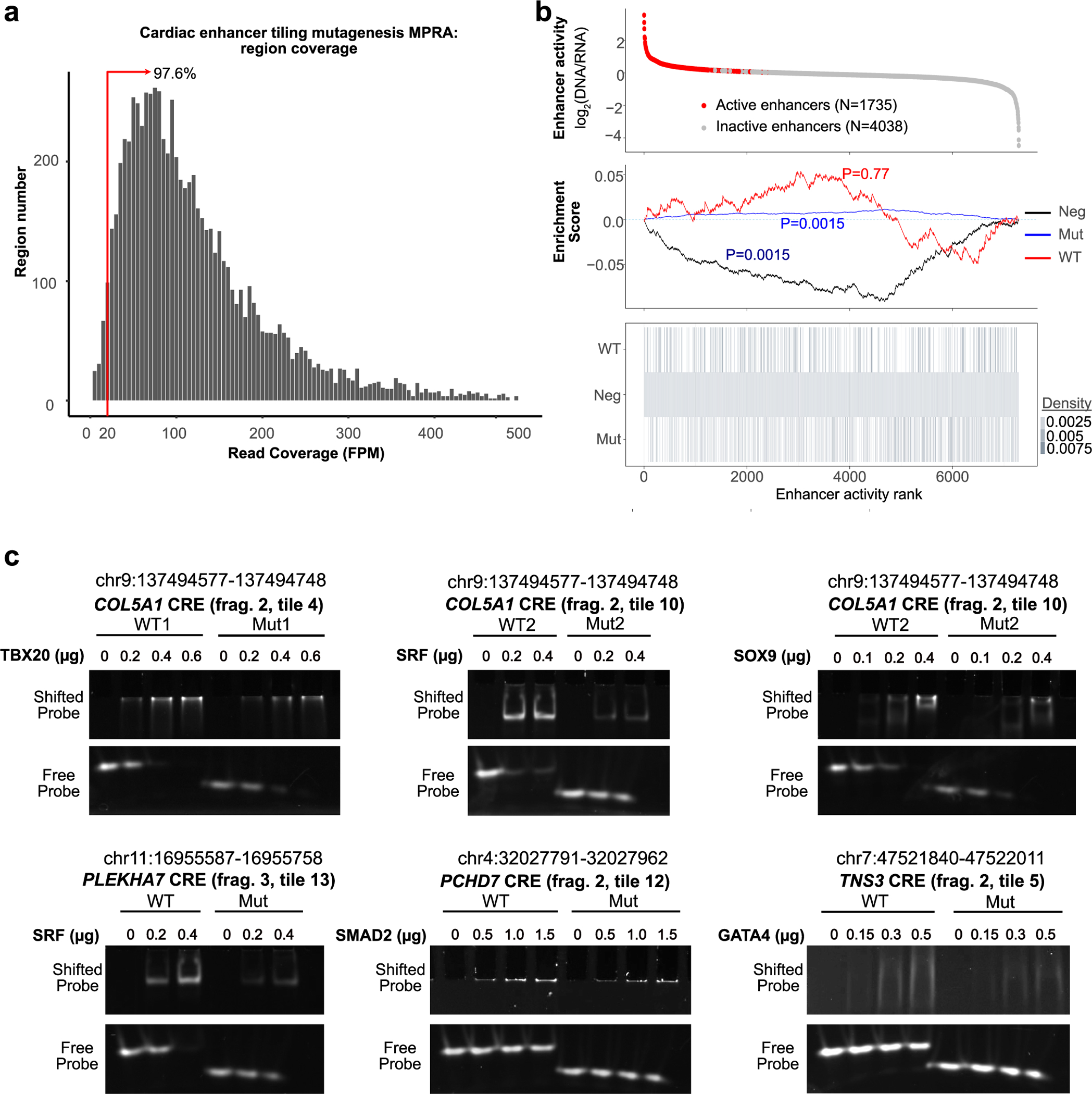
Functional dissection of active cardiac enhancers by tiling deletion mutagenesis. **a**. Coverage of designed regions. Red line shows minimum coverage for inclusion in analysis (FPM ≥ 20). 97.6% of regions had coverage ≥20 FPM. **b**. Summary of activity of regions in the mutagenesis MPRA. Line plot at the bottom shows a vertical line for each tested region with the indicated annotation. Enrichment score indicates how the indicated annotations are distributed across the regions, ranked by activity. Enrichment p-value with Bonferroni correction was calculated using a 1-sided permutation test (see Methods). Active enhancers had barcodes that were overrepresented in RNA compared to DNA (DESeq *P*_adj_ < 0.05). **c**. Validation of effects of mutations on transcription factor binding. Transcription factor binding was evaluated by electrophoretic mobility shift assay. The indicated wild-type and mutant oligonucleotide pairs were incubated with transcription factors with predicted altered motifs and analyzed by gel electrophoresis. Results are representative of at least 2 independent experiments.

**Fig. 2 |:**
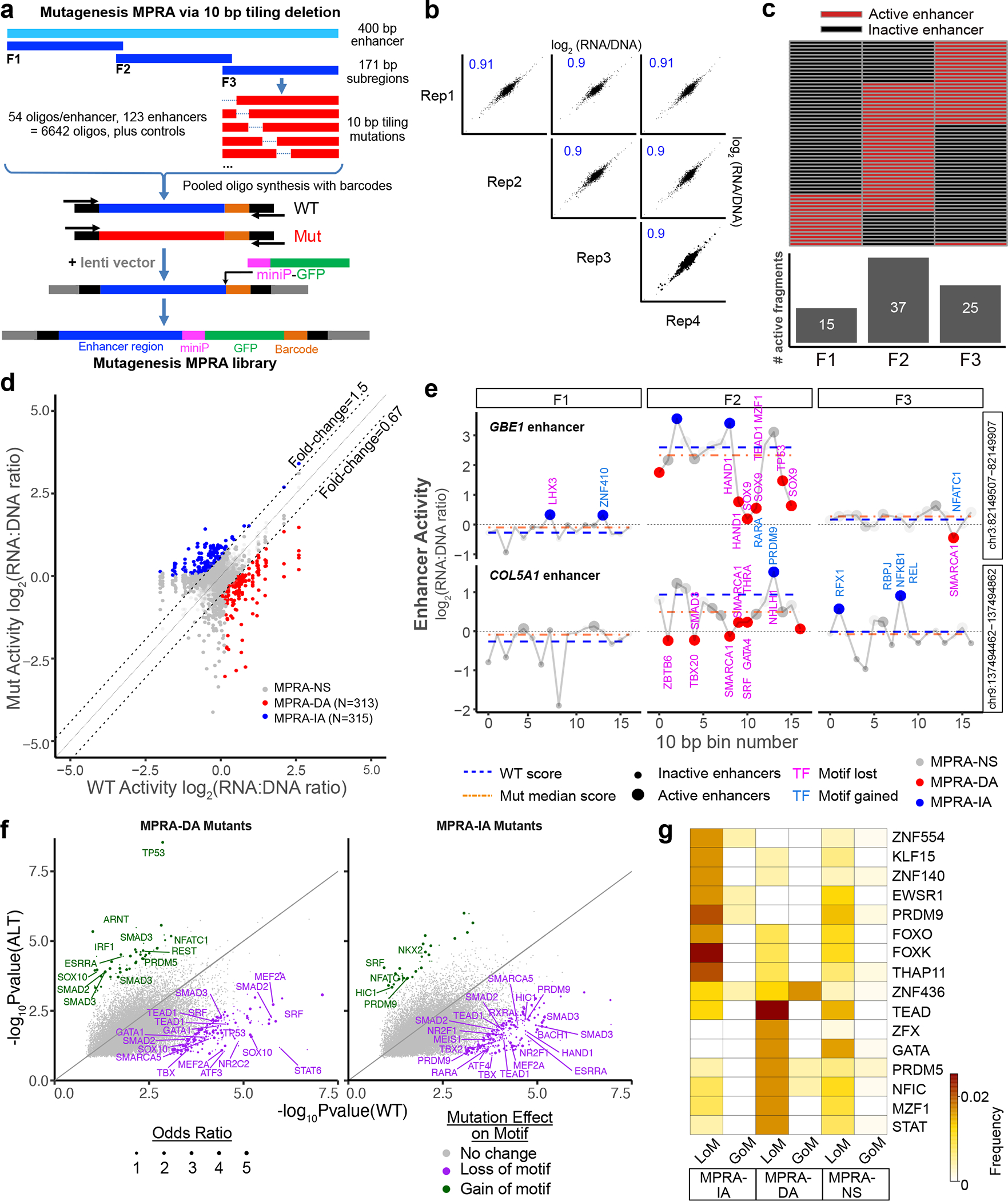
Tiling deletion analysis of human cardiac enhancers. Systematic tiling mutagenesis was performed on 123 active cardiac enhancers using the lentiMPRA/iPSC-CM platform. **a**, Design of mutagenesis MPRA. Each original 400 bp enhancer was divided into three 171 bp subregions (F1–F3), and each subregion was tiled with 10 bp deletions. The barcoded oligos were inserted into a lentiMPRA vector so that the barcode was in the reporter gene’s 3′ UTR. **b**, Reproducibility of mutagenesis MPRA. Four independent replicates were obtained on iPSC-CM culture day 24. Replicate samples were highly correlated. Pearson correlation is shown. **c**, Summary of activity of wild-type enhancer subregions. Each line represents the three subregions of an active 400 bp enhancer. **d**, Summary of mutagenesis MPRA results. Dashed diagonal lines indicate 50% fold change (FC) thresholds. Each point represents one wild-type–mutant (WT–Mut) pair. Sequences in which the members of the pair had different activity (FC ≥ 1.5, *P*_adj_ < 0.05, at least one member of pair active) are colored. MPRA-DA, MPRA-IA and MPRA-NS indicate that the mutation decreased, increased or did not change MPRA activity, respectively, compared to WT. *P*_adj_ was calculated using two-way paired *t*-tests with Benjamini–Hochberg correction. **e**, Representative example of mutagenesis data for the GBE1 and COL5A1 enhancers. Activity of a wild-type sequence and the median of its mutant counterpart are shown by dashed blue and orange lines, respectively. Larger circles indicate sequences with detectable activity. Colors indicate a significant change of activity in the mutant sequence compared to the wild-type pair. Transcription factor motifs created or ablated by mutation are shown in magenta and blue, respectively. **f**, Summary motif analysis of tiling mutagenesis. Each point represents one motif family and one WT–Mut pair. Motif significance scores are nominal *P* values reported by FIMO^39^. Colored points indicate that a Mut sequence lost or gained a motif compared to its wild-type counterpart. The size of each point represents the odds ratio that the motif was changed compared to MPRA-NS. Complete table of results can be found in Supplementary Data 2. **g**, Top transcription factor motifs, ranked by frequency.

To further define the sequence features of active cardiac enhancers, we next performed systematic, tiling mutagenesis of the top 123 cardiac enhancers identified by lentiSTARR-seq. For these studies of enhancer variants, we used a lentiMPRA design in which test sequences were positioned upstream of a minimal promoter–reporter, and a short barcode was placed in the 3′ UTR (Fig. 2a). This design correlated well with other MPRA designs^20^, and the barcode facilitates the identification of enhancer variants. Because of barcode ‘hopping’ between variants with largely similar sequences, we avoided self-priming PCR and instead represented each 400 bp region as three fragments (F1, F2 and F3), each containing 171 bp of genomic sequence (Fig. 2a). These reference sequences were then tiled with 17 (10-bp) deletions, and each sequence was uniquely barcoded and flanked with primer binding sites (Supplementary Data 2). Oligonucleotides were synthesized as a pool and cloned into the lentiMPRA vector. The packaged lentiviral library was applied to iPSC-CMs on differentiation day 17. A week later, the barcoded 3′ UTR amplicon was amplified from RNA or genomic DNA and sequenced (Supplementary Data 2). Regions with insufficient coverage (fragments per million (FPM) < 20) were excluded from further analysis (2.4% of regions; Extended Data Fig. 3a). Four independent replicates showed excellent correlation (Pearson *r* > 0.9; Fig. 2b). Of the 123 initial 400 bp active enhancers, 59 exhibited activity in at least one 171 bp reference fragment (Fig. 2c and Extended Data Fig. 3b), with activity most often contained in the central (F2) fragment (Fig. 2c), which overlapped the ATAC-seq peak center. Analysis of each reference fragment and its associated mutants identified 628 (10-bp) deletions that significantly affected enhancer activity (Fig. 2d). Notably, half (313) decreased activity (MPRA-DA) and half (315) increased activity (MPRA-IA).

To gain insights into how these mutations influenced enhancer activity, we analyzed transcription factor binding motifs in reference and mutant sequences (Supplementary Data 2), as exemplified for enhancers adjacent to *GBE1* and *COL5A1* (Fig. 2e). Tiled mutations in the active F2 fragment of the *GBE1* enhancer reduced its activity and abolished TEAD1, MZF1, SOX9 and HAND1 motifs (loss-of-motif (LoM)) and generated a new RARA motif (gain-of-motif (GoM); Fig. 2e (top)). For the active F2 fragment of the *COL5A1* enhancer, the elimination of GATA4, SRF, SMAD3, THRA and TBX20 motifs reduced enhancer activity, whereas a deletion that created a PRDM9 motif increased enhancer activity (Fig. 2e (bottom)). To systematically identify motifs that reduced or increased enhancer activity when eliminated or created, we scanned each MPRA-DA or MPRA-IA reference-mutant pair for each transcription factor motif to identify significantly impacted motifs (Fig. 2f). Among these motifs were several belonging to transcription factors that regulate heart development, such as TBX, GATA, SRF and SMAD. Motifs with less clear involvement in cardiomyocyte development, such as SOX, NFAT, PRDM and TP53, were also identified. We validated the effects of mutations on the binding of TBX20, SRF, SMAD2, SOX9 and GATA4 using the electrophoretic mobility shift assay (EMSA; Extended Data Fig. 3c). To identify the motifs most linked to changes in enhancer activity across the experiment, we calculated the frequency that each motif was perturbed by MPRA-IA or MPRA-DA mutations, compared to mutations that did not affect enhancer activity (Fig. 2g). Loss of TEAD, GATA and PRDM5 motifs was among the most frequently linked to reduced enhancer activity, whereas loss of FOXK and PRDM9 motifs was most frequently associated with increased enhancer activity.

Together, the tiling mutagenesis showed that the lentiMPRA platform robustly detects the effect of sequence variants on enhancer activity and identified transcription factor motifs important for cardiac enhancer activity.

### Analysis of CHD ncDNVs for effect on cardiac CRE activity

**Extended Data Fig. 4 |:**
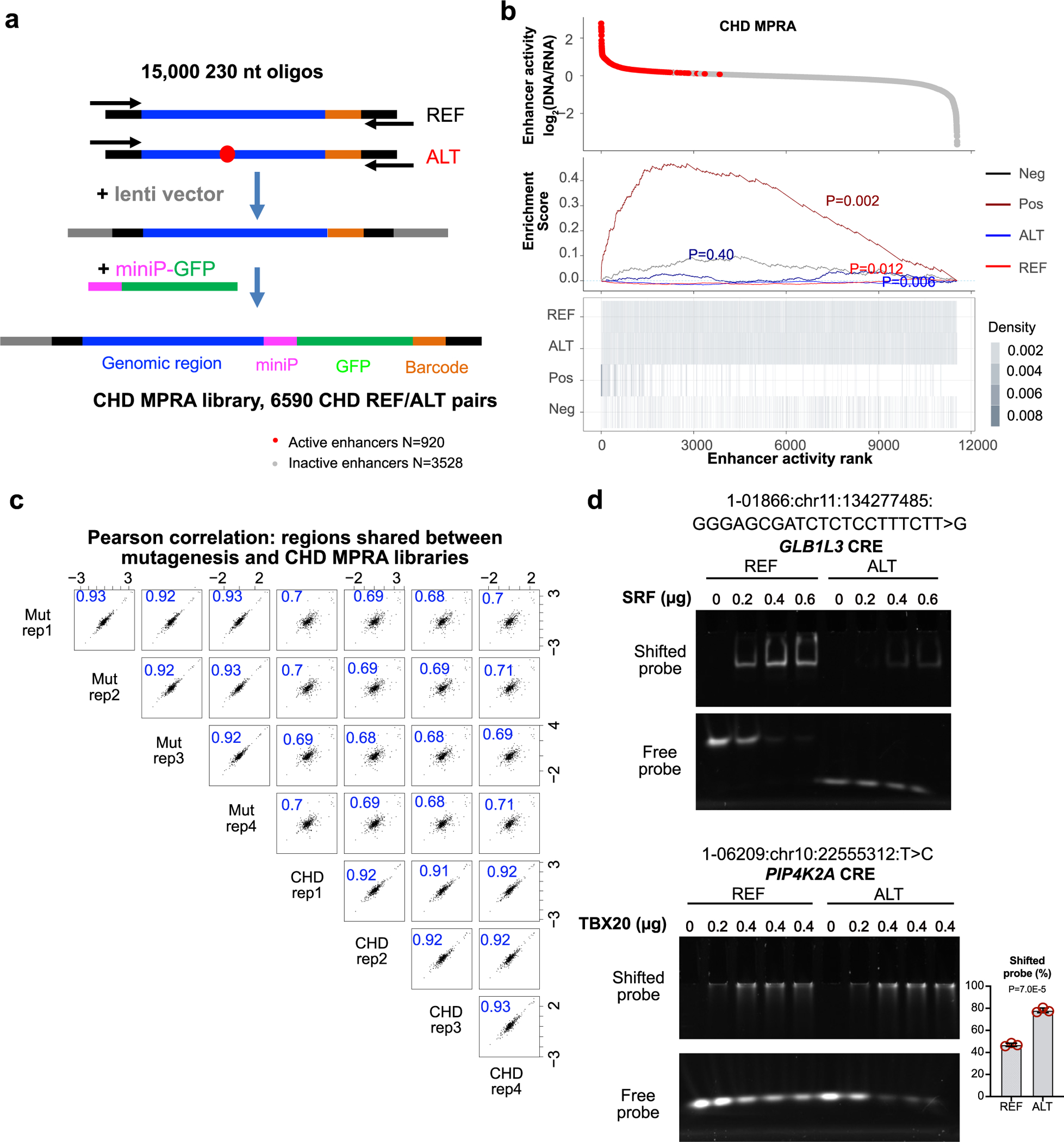
CHD MPRA library characterization. **a**. The CHD MPRA library included 6590 REF-ALT pairs. After pooled library synthesis of barcoded oligos, the oligos were PCR amplified and cloned into lentivirus genome backbone. A minimal promoter (miniP)-GFP cassette was then inserted into the cloned oligo library. **b**. Summary of activity of CHD MPRA library. Plot on bottom indicates the occurrence of the indicated annotation with a vertical line. Enrichment score represents enrichment of the indicated set of annotations at either end of the list of all regions, ranked by activity. Enrichment p-value was determined by 1-sided permutation test, with Bonferroni correction. Active enhancers had barcodes overrepresented in RNA compared to DNA (DESeq2 *P*_adj_ < 0.05). **c**. Pearson correlation (PCC) between regions shared between the Mutagenesis MPRA and the CHD MPRA. The same genomic sequences had different barcodes in the two assays. **d**. Validation of the effect of variants on transcription factor binding. EMSA assay was used to test the binding of SRF or TBX20 to REF or ALT variant sequences. For the GLB1L3 CRE, ALT disrupted the SRF motif and reduced SRF binding in the EMSA assay. For the PIP4K2A CRE, ALT generated a TBX20 motif and increased TBX20 binding in the EMSA assay. Representative of three independent experiments. Two-tailed t-test. n = 3 per group. Graph shows mean ± SD.

**Fig. 3 |:**
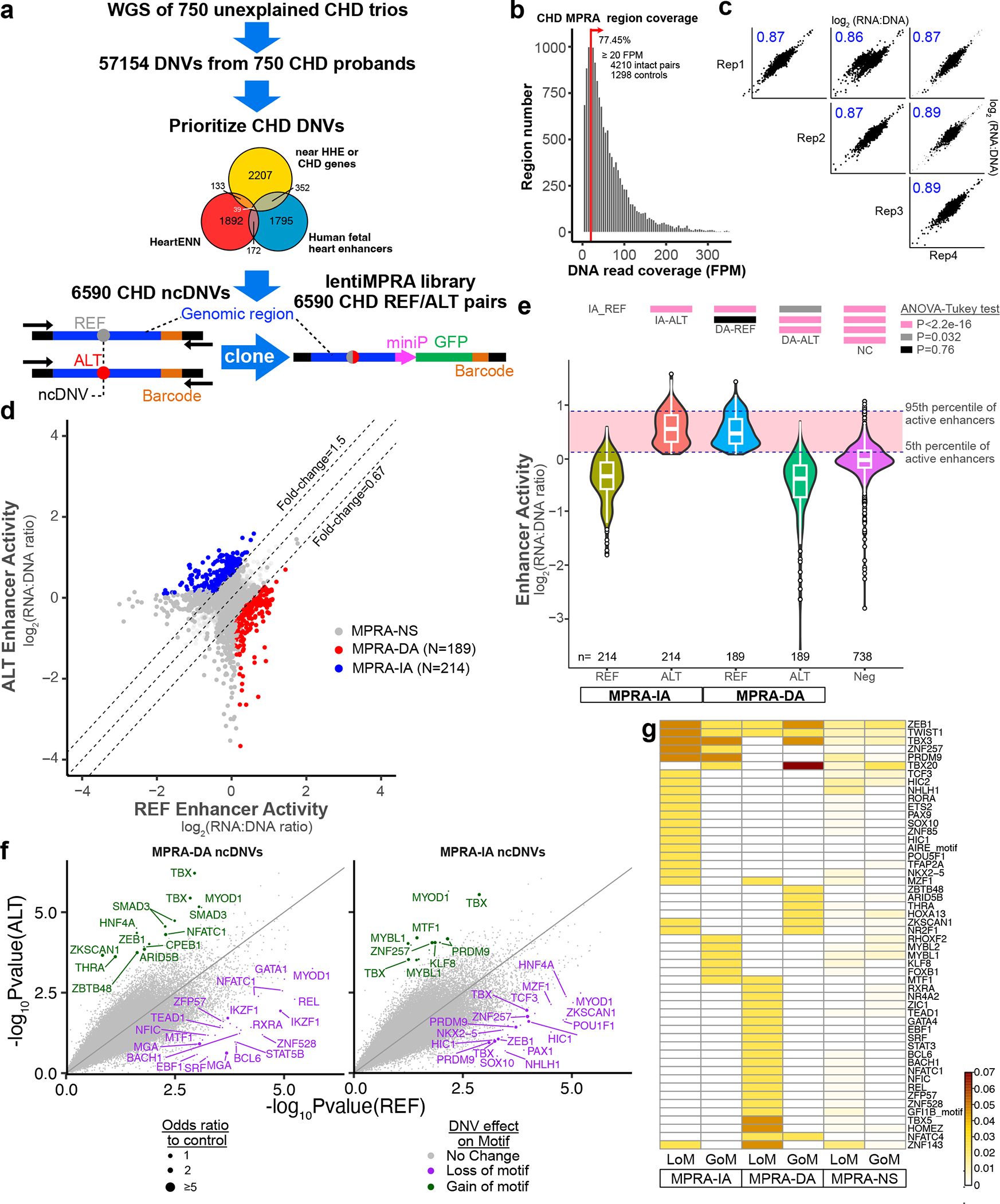
Dissection of CHD ncDNV impact on cardiac enhancer activity. **a**, CHD ncDNV prioritization. The 6,590 prioritized ncDNVs were each synthesized as a REF and ALT pair of 230 nt oligos, in which a 171 bp genomic region was centered on the ncDNV. Barcoded oligos were cloned into the lentiMPRA as depicted for the mutagenesis MPRA in Fig. 2a. **b**, Histogram showing coverage of designed regions. Red line shows minimum coverage in amplicons from genomic DNA for inclusion in analysis (FPM ≥ 20). **c**, Reproducibility of CHD lentiMPRA. Four independent replicates were obtained on iPSC-CM culture day 24. There was high correlation (Pearson *r* > 0.86) between replicates. **d**, Summary of CHD MPRA results. Dashed diagonal lines indicate 50% fold change thresholds. Each point represents a ncDNV’s REF–ALT pair. Colored points indicated differential activity between REF and ALT (two-way paired *t*-test with BH correction <0.05; |log_2_(FC)| >0.58; active in at least one replicate). **e**, Effect of MPRA-DA and MPRA-IA ncDNVs on enhancer activity. In MPRA-DA regions, REF exhibited enhancer activity and overall ALT had negligible activity. In MPRA-IA regions, REF had negligible activity and ALT had enhancer activity comparable to REF in MPRA-DA regions. Dotted lines indicate the 5th and 95th percentile values of active enhancers. Statistical comparison by ANOVA with the Tukey post hoc test is shown above the plot. Numbers at the bottom of the plot indicate number of regions in each group. Center, box and whiskers indicate median, 25th and 75th percentiles and value closest to 25th percentile minus or 75th percentile plus 1.5 times the interquartile range. **f**, Effect of ncDNVs on transcription factor motifs in MPRA-DA and MPRA-IA regions. See Fig. 2f for details. Complete table of results can be found in Supplementary Data 3. **g**, Top transcription factor motifs impacted by ncDNVs, ranked by frequency.

We next deployed lentiMPRA to analyze the contribution of ncDNVs to CHD. We hypothesized that a subset of ncDNVs contributes to CHD pathogenesis by altering cardiac CRE activity. From WGS of 750 CHD trios who did not have a putative identified genetic etiology, we prioritized 6,590 ncDNVs from 57,154 DNVs based on annotation as noncoding, chromatin features, proximity to genes with high heart expression^2^ or implicated in CHD and previously described bioinformatic approaches^6^, of which 89.9% were single-nucleotide variants (Fig. 3a, Extended Data Fig. 4a, Supplementary Table 3, Supplementary Data 3 and Methods). The MPRA library included a median of eight ncDNVs per participant (interquartile range of 6–11). Each prioritized ncDNV was represented by a reference (REF) and variant (ALT) pair, comprising 171 bp of genomic sequence centered on the ncDNVs (Fig. 3a, Extended Data Fig. 4a and Supplementary Data 3). We included 865 negative controls (ATAC-seq peaks in iPS cells and not iPSC-CMs), 217 positive controls (regions with enhancer activity in the mutagenesis MPRA) and 396 additional controls from the mutagenesis MPRA. The resultant 15,000 pooled oligos were synthesized following the same barcoded design as used for tiling mutagenesis. Day 17 iPSC-CMs were treated with the resulting lentiviral library. We quantified enhancer activity from barcode frequency in RNA compared to genomic DNA on day 24 (Extended Data Fig. 4b and Supplementary Data 3). The library had sufficient coverage of 77.5% of regions (FPM ≥ 20; 4,210 intact REF–ALT pairs; Fig. 3b), and four biological replicates were well correlated (Pearson *r* > 0.86; Fig. 3c). Control oligos shared between the mutagenesis MPRA and the CHD MPRA libraries were highly correlated despite having different barcodes (*r* = 0.69; Extended Data Fig. 4c), underscoring assay reproducibility and indicating that specific barcode sequences are not major activity determinants.

A total of 1,835 regions exhibited enhancer activity, 771 only in the REF allele, 769 only in the ALT allele and 295 in both alleles. A total of 403 ALT–REF pairs differed significantly in activity. Of these, 214 ncDNVs (195 single-nucleotide variants and 19 indels from 183 participants) increased enhancer activity (MPRA-IA) and 189 ncDNVs (174 single-nucleotide variants and 15 indels from 170 participants) decreased enhancer activity (MPRA-DA; Fig. 3d). The remaining ncDNVs that did not significantly affect enhancer activity were designated MPRA-NS. Overall, the REF allele of MPRA-DA regions had enhancer activity, and the corresponding ALT allele had negligible activity comparable to negative controls (Fig. 3e). By contrast, the ALT allele of MPRA-IA regions had enhancer activity, whereas the corresponding REF allele had negligible activity (Fig. 3e). These results suggest that MPRA-IA ncDNVs confer new enhancer activity to REF sequences. The level of activity of the created enhancers was comparable to that of endogenous enhancers.

We analyzed transcription factor binding motifs changed by MPRA-IA and MPRA-DA ncDNVs (Supplementary Data 3). MPRA-DA ncDNVs often caused loss of transcription factor motifs linked to heart development, including MGA/T-box, TEAD1, SRF and GATA motifs, and MPRA-IA ncDNVs most frequently had gain or loss of T-box, E-box (for example, ID4 and MYOD1) and PRDM9 motifs (Fig. 3f,g). The effect of an MPRA-IA and an MPRA-DA ncDNV on transcription factor DNA binding was validated by EMSA (Extended Data Fig. 4d).

### CHD gene-associated functional ncDNV effect on iPSC-CMs

**Extended Data Fig. 5 |:**
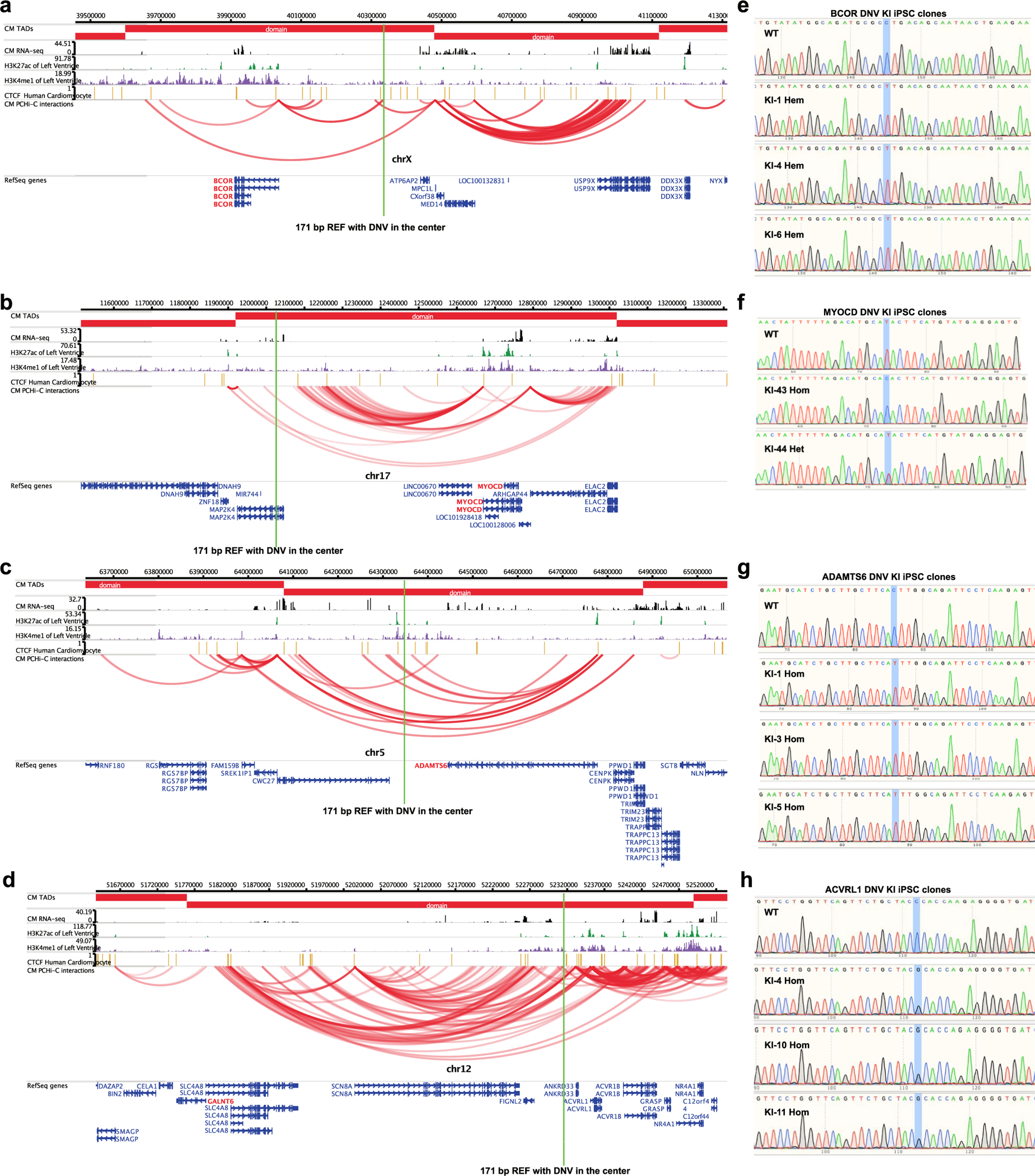
Genomic loci of CHD-associated ncDNVs. **a**–**d**. WashU Epigenome Browser views of loci containing 4 ncDNVs. Promoter capture Hi-C and RNA-seq in iPSCs and iPSC-CMs from ref. 33, PMID 29988018. Genes dysregulated by DNVs are indicated in red. Green lines highlight 171 bp REF region with DNV in the center. **e**–**h**. Sanger sequencing traces of genome edited iPSC lines.

**Fig. 4 |:**
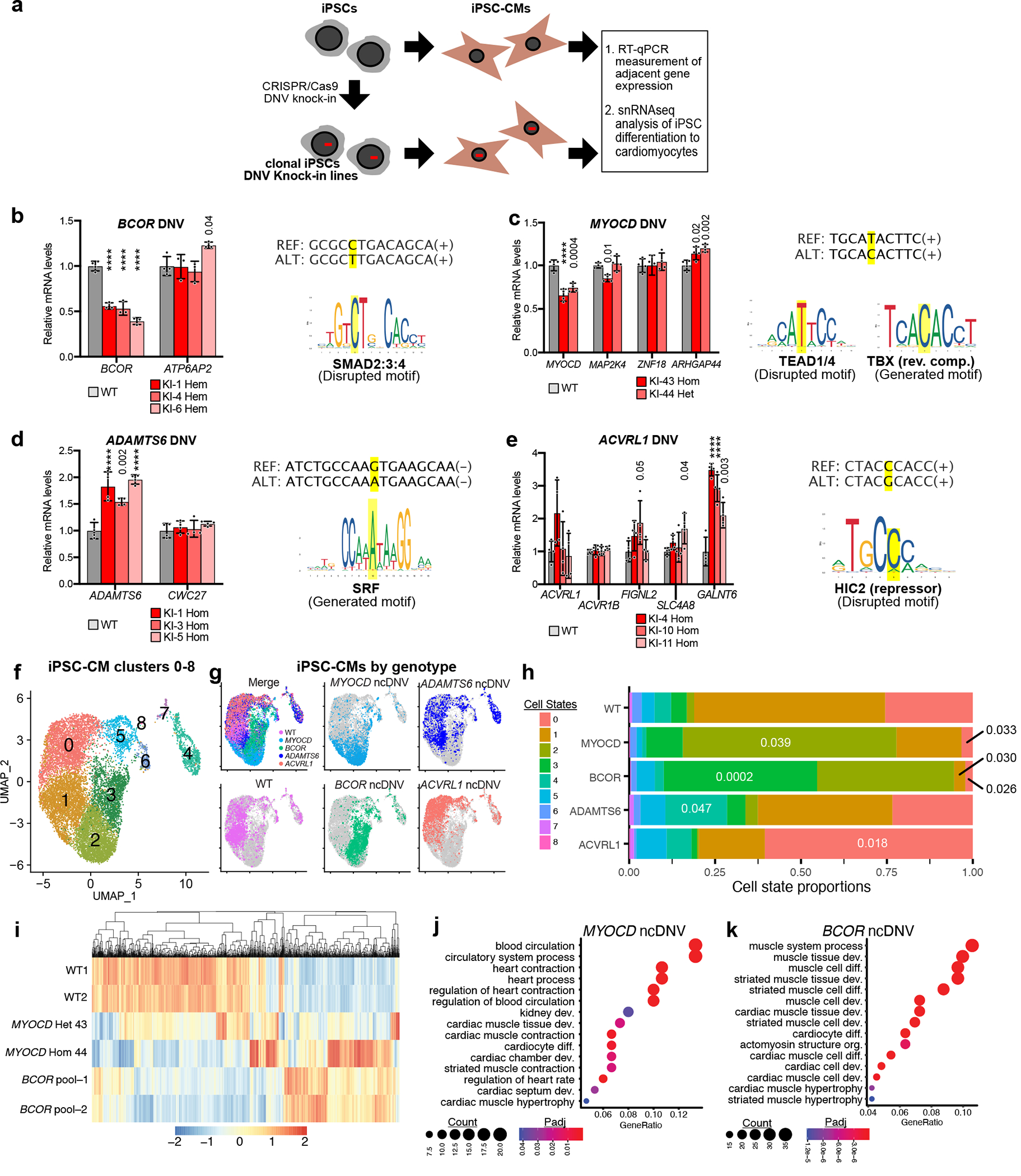
Characterization of CHD gene-associated ncDNVs in iPSC-CMs. **a**, Schematic representation of characterization of CHD ncDNVs in iPSC-CMs. CRISPR–Cas9 was used to introduce ncDNVs from CHD lentiMPRA into their endogenous loci. After isolation of clonal lines and differentiation to iPSC-CMs, the expression of neighboring genes was measured by RT–qPCR. **b**–**e**, Validated ncDNVs and their impact on neighboring CHD-associated genes. Bar plots show RT–qPCR analysis of genes adjacent to DNVs near *BCOR* (**b**), *MYOCD* (**c**), *ADAMTS6* (**d**), and *ACVRL1* (**e**) in day 17 iPSC-CMs. Data are shown as mean ± s.d. of at least three independent experiments. ANOVA with Dunnett’s test compared to wild-type (WT) control. REF and ALT sequences are shown to the right, with the SNV highlighted in yellow, along with predicted transcription factor binding motifs impacted by SNVs. *BCOR* knock-in lines, *n* = 3. All others, *n* = 4. **f**,**g**, UMAP projection of wild-type and ncDNV knock-in nuclei from iPSC-CMs at day 10 of differentiation. **f**, iPSC-CM clusters are colored and numbered 0–8. **g**, Nuclei are colored by genotype. Merge (top-left) shows all genotypes with indicated colors and the remaining panels each show one genotype. **h**, Stacked bar graph of the percentages of nuclei in each cluster. The proportion of nuclei in each cluster was compared to wild-type nuclei; numbers indicate significant *P* values (one-way ANOVA with Dunnett’s multiple comparison test). **i**, Heatmap of genes that were differentially expressed in *BCOR* or *MYOCD* ncDNV knock-ins compared to wild type. Genes that were significantly different from wild-type in both replicates (Seurat FindMarkers *P*_adj_ < 0.05; Methods) were selected. Heatmap displays the scaled average gene expression from each replicate. **j**,**k**. Gene Ontology analysis of the genes differentially expressed in both *MYOCD* ncDNV homozygous and heterozygous lines (**j**) or in both *BCOR* ncDNV lines (**k**) compared to wild-type iPSC-CMs. Hypergeometric test with Bonferroni correction for multiple testing. In **b**–**e**, ****P < 0.0001.

To assess their impact in their endogenous genomic context, we introduced seven MPRA-DA and three MPRA-IA ncDNVs into iPS cells by CRISPR–Cas9 genome editing (Fig. 4a and Supplementary Table 4). These ncDNVs were selected to neighbor a known CHD gene, to be in or adjacent to a promoter–enhancer loop anchor and to be readily modified by CRISPR–Cas9 genome editing (Extended Data Fig. 5a–d and Supplementary Data 3). We isolated two to five independent, isogenic, clonal lines for each ncDNV (Extended Data Fig. 5e–h) and differentiated each line into iPSC-CMs at least three independent times. We then measured the expression of genes neighboring each ncDNV by qRT–PCR. Four of ten CHD ncDNVs significantly and reproducibly altered the expression of the neighboring gene(s) (Fig. 4b–e and Supplementary Table 4). Six ncDNVs that impacted enhancer activity by MPRA did not measurably affect neighboring gene expression in day 17 iPSC-CMs. These ncDNVs may be functionally important in other biological contexts or the regulation of other genes. We also cannot exclude redundant CREs that mask functional impact in this assay.

**Extended Data Fig. 6 |:**
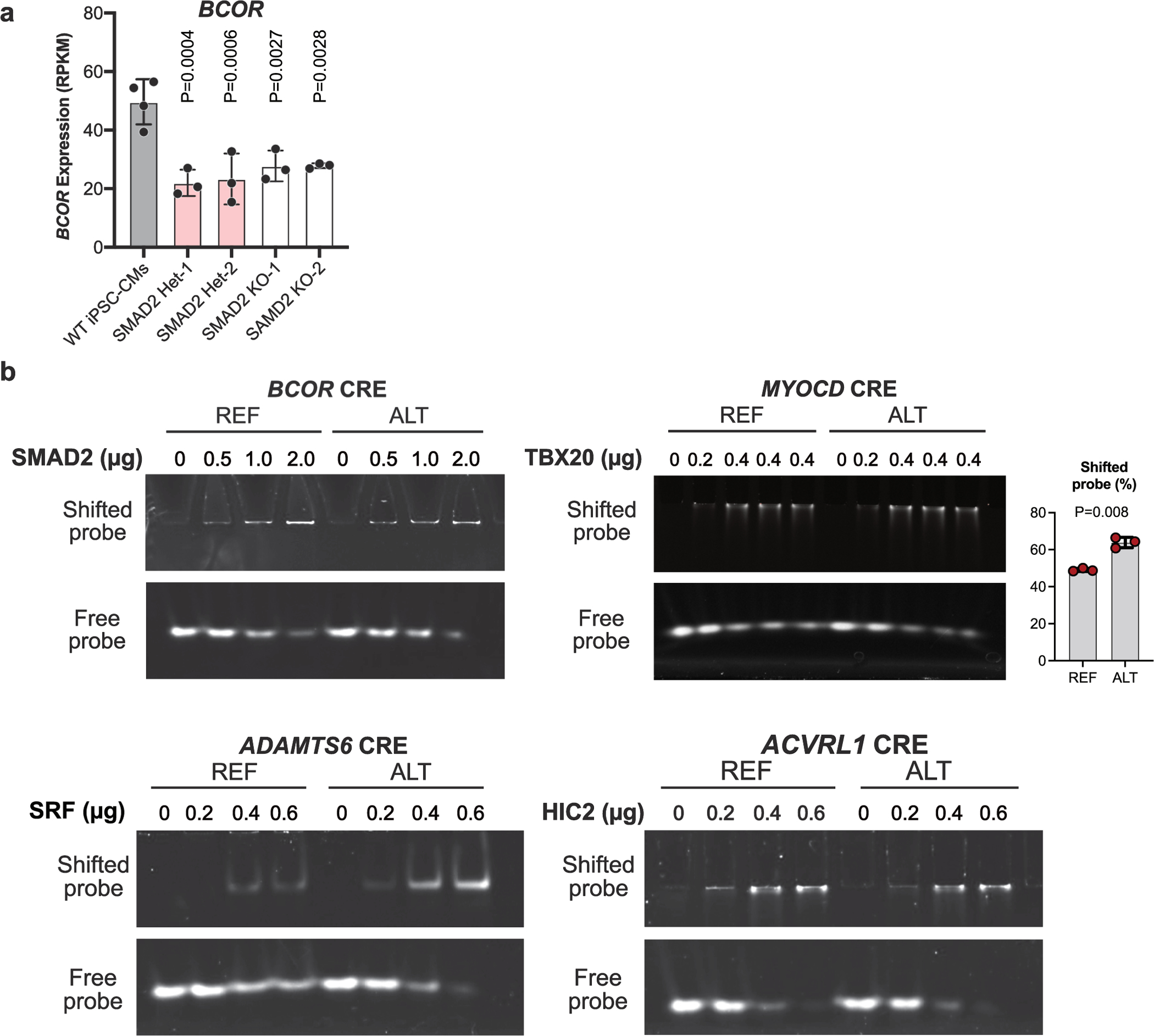
Characterization of iPSC-CMs with knockin of CHD gene-associated noncoding DNVs. **a**. *BCOR* downregulation in *SMAD2* Het and KO iPSC-CMs. Gene expression was measured by RNA-seq. One-way ANOVA with Dunnett’s multiple comparison test versus WT. n = 3. **b**. Effect of ncDNVs on binding of transcription factors to CREs near CHD genes. 39 bp duplexes centered on ncDNVs neighboring 4 CHD genes were synthesized. Binding of purified, recombinant proteins to the REF or ALT sequence was measured by electrophoretic mobility shift assay (EMSA). SMAD2 and HIC2 bound CREs near *BCOR* and *ACVRL1* more strongly for REF compared to ALT. In contrast, SRF and TBX20 bound CREs near *ADAMTS6* and *MYOCD* more strongly for ALT compared to REF. Note lower free probe in *MYOCD*-ALT compared to REF. Results are representative of at least three independent experiments. Quantification of TBX20 EMSA: mean ± SD; n = 3; two-sided t-test. Graphs in **a** and **b** show mean ± SD.

Two MPRA-DA ncDNVs reduced the expression of adjacent CHD genes *BCOR* and *MYOCD*, respectively (Fig. 4b,c). BCOR, a BCL-6 corepressor, is part of a transcriptional repression complex. Mutations in *BCOR* cause oculofaciocardiodental syndrome, an X-linked dominant, male lethal condition that includes cardiac septal defects^22,23^. The adjacent ncDNV occurred in a female patient with atrial septal defect and hypoplastic left heart syndrome. Introduction of this ncDNV into the endogenous locus downregulated *BCOR* in three independent iPSC-CM lines (Fig. 4b). The ncDNV disrupted a SMAD binding motif in a distal intergenic region (Fig. 4b) that interacts with the BCOR promoter in iPSC-CMs (Extended Data Fig. 5a). We confirmed that *BCOR* was downregulated in both *SMAD2*^−/−^ and *SMAD2*^*+*/−^ iPSC-CMs (Extended Data Fig. 6a). Moreover, the variant weakened DNA binding by SMAD2 (Extended Data Fig. 6b).

MYOCD activates cardiac muscle promoters by associating with SRF, which is required for heart development and cardiomyocyte differentiation^24,25^. Human *MYOCD* mutations cause CHD and megabladder^26^. The neighboring ncDNV was within an intron *MAP2K4* and close to a chromatin loop anchor that contacts the *MYOCD* promoter (Extended Data Fig. 5b). This ncDNV disrupted a potential TEAD binding motif and concurrently installed a TBX binding motif (Fig. 4c and Extended Data Fig. 6b). Genome editing yielded two independent iPS cell lines, one heterozygous and one homozygous for the ncDNV at the endogenous locus. In both mutant lines, iPSC-CMs expressed lower levels of *MYOCD* (Fig. 4c). In the homozygous line, *MAP2K4* was also moderately but significantly downregulated (Fig. 4c).

We also validated two MPRA-IA ncDNVs, which increased the expression of *ADAMTS6* and *GALNT6*, respectively (Fig. 4d,e and Extended Data Fig. 5c,d). ADAMTS6 is a metalloprotease that mediates extracellular proteolysis of extracellular matrix components and other secreted molecules^27^. *Adamts6*-null mice developed embryonic heart defects including double outlet right ventricle, atrioventricular septal defect and ventricular hypertrophy^28^. The ncDNV, located near an *ADAMTS6* promoter loop (Extended Data Fig. 5c), created a new SRF binding motif and upregulated *ADAMTS6* expression in three independent homozygous iPSC-CM lines (Fig. 4d and Extended Data Fig. 6b).

A MPRA-IA ncDNV that impacted *GALNT6* was initially selected because it neighbors *ACVRL1*, a known CHD gene (Extended Data Fig. 5d). iPSC-CMs derived from three independent iPS cell lines with homozygous knock-in of this ncDNV had unperturbed *ACVRL1* expression but significantly upregulated *GALNT6* (Fig. 4e), a glycosyltransferase responsible for the initiation of mucin-type O-glycosylation. *GALNT1*, a *GALNT6* paralog, is required for mouse heart development and function^29^, suggesting a potential role of *GALNT6* in cardiac development. This ncDNV, located within a loop anchor that contacts the *GALNT6* promoter (Extended Data Fig. 5d), disrupted the motif of transcriptional repressors HIC1 and HIC2 in an intergenic region that interacts with the *GALNT6* promoter (Extended Data Fig. 6b), plausibly explaining the upregulation of *GALNT6*. HIC2 is required for normal heart development, and its haploinsufficiency may contribute to cardiac defects observed in 22q11 deletion syndrome^30^.

**Extended Data Fig. 7 |:**
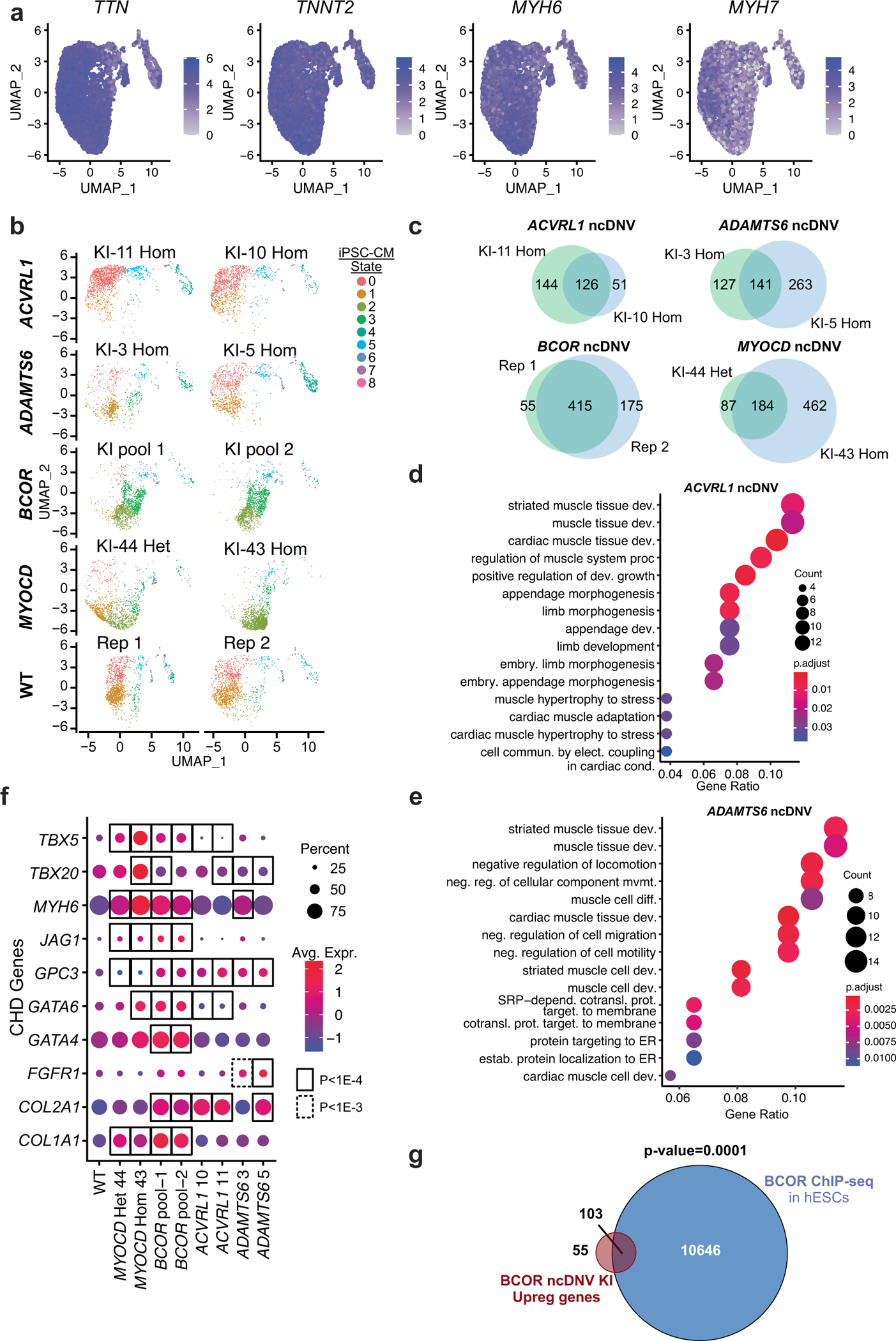
snRNA-seq characterization of the impact of four ncDNVs that impact MPRA activity on iPSC differentiation to iPSC-CMs. **a**. Expression of cardiac marker genes. Most nuclei contained cardiomyocyte marker genes. **b**. Two independent iPSC clones per ncDNV (ACVRL1, ADAMTS6, MYOCD) or knockin pools (BCOR) were separately differentiated into iPSC-CMs and then analyzed by multiplexed snRNA-seq. After clustering, UMAP plots of individual cells are shown separately for each independent differentiation. **c**–**e**. Pseudo-bulk differential gene expression analysis. The number of differentially expressed genes for each independent replicate vs. wild type was analyzed from snRNA-seq data. Differentially expressed genes for the two replicates showed excellent overlap (**c**). Gene ontology terms enriched in differentially expressed genes shared between biological replicates for *ACVRL1* ncDNV KI lines (**d**) or *ADAMTS6* ncDNV KI lines (**e**). BH-corrected hypergeometric p-values. **f**. CHD genes differentially expressed in iPSC-CMs containing indicated ncDNV knockins compared to wild-type (WT). The selected CHD genes were mouse or human CHD genes (see Supplementary Data 5) that overlapped with genes differentially expressed in both replicates of any of the four introduced ncDNVs. BH-corrected P values were reported by Seurat FindMarkers function. **g**. Comparison of genes upregulated in BCOR ncDNV KI pool iPSC-CMs compared to BCOR genome occupancy in H1 hESCs (GSE104690). One-sided permutation test (10000 permutations).

To further assess the impact of these four ncDNVs on cardiomyocyte differentiation, we analyzed early iPSC-CMs (differentiation day 10) using single-nucleus RNA-seq (snRNA-seq). For each ncDNV near *MYOCD*, *ADAMTS6* and *ACVRL1*-*GALNT6*, we analyzed two independent clonal knock-in lines, each differentiated separately. For the *BCOR* ncDNV, we analyzed two independent differentiations of the polyclonal pool of CRISPR–Cas9 genome editing, because editing of the X-linked *BCOR* locus was highly efficient (Supplementary Table 4), and we observed waning effects of the *BCOR* ncDNV on *BCOR* expression with iPS cell passage. To minimize the batch effect, nuclei from separate differentiations were each labeled with a distinct barcode and then pooled for snRNA-seq library preparation and sequencing^31^. Analysis of nuclear transcriptomes identified nine cell states that expressed cardiomyocyte markers (CM0–CM8; Fig. 4f and Extended Data Fig. 7a). The replicate clonal lines differentiated into similar cell state patterns (Extended Data Fig. 7b). The parental wild-type cell line primarily yielded CM0 and CM1. This pattern was significantly altered by four impactful ncDNVs—the *MYOCD*, *BCOR* and *ADAMTS6* ncDNVs significantly expanded CM2, CM3 and CM4 populations, respectively, whereas the *ACVRL1*-*GALNT6* ncDNV significantly expanded CM0 (Fig. 4g,h). Differentially expressed genes in the ncDNV knock-ins were functionally related to muscle and cardiac cell differentiation and development, cell migration and blood circulation (Fig. 4i–k and Extended Data Fig. 7c–e) and included several established CHD genes, such as *GATA4*, *GATA6* and *TBX5* (Extended Data Fig. 7f). Upregulation of *GATA4* and *GATA6* in the *BCOR* ncDNV knock-in pool was particularly intriguing because these genes are directly repressed by BCOR^32^; indeed, 65% of genes upregulated in the *BCOR* ncDNV knock-ins were enriched for BCOR binding^32^ (Extended Data Fig. 7g).

**Extended Data Fig. 8 |:**
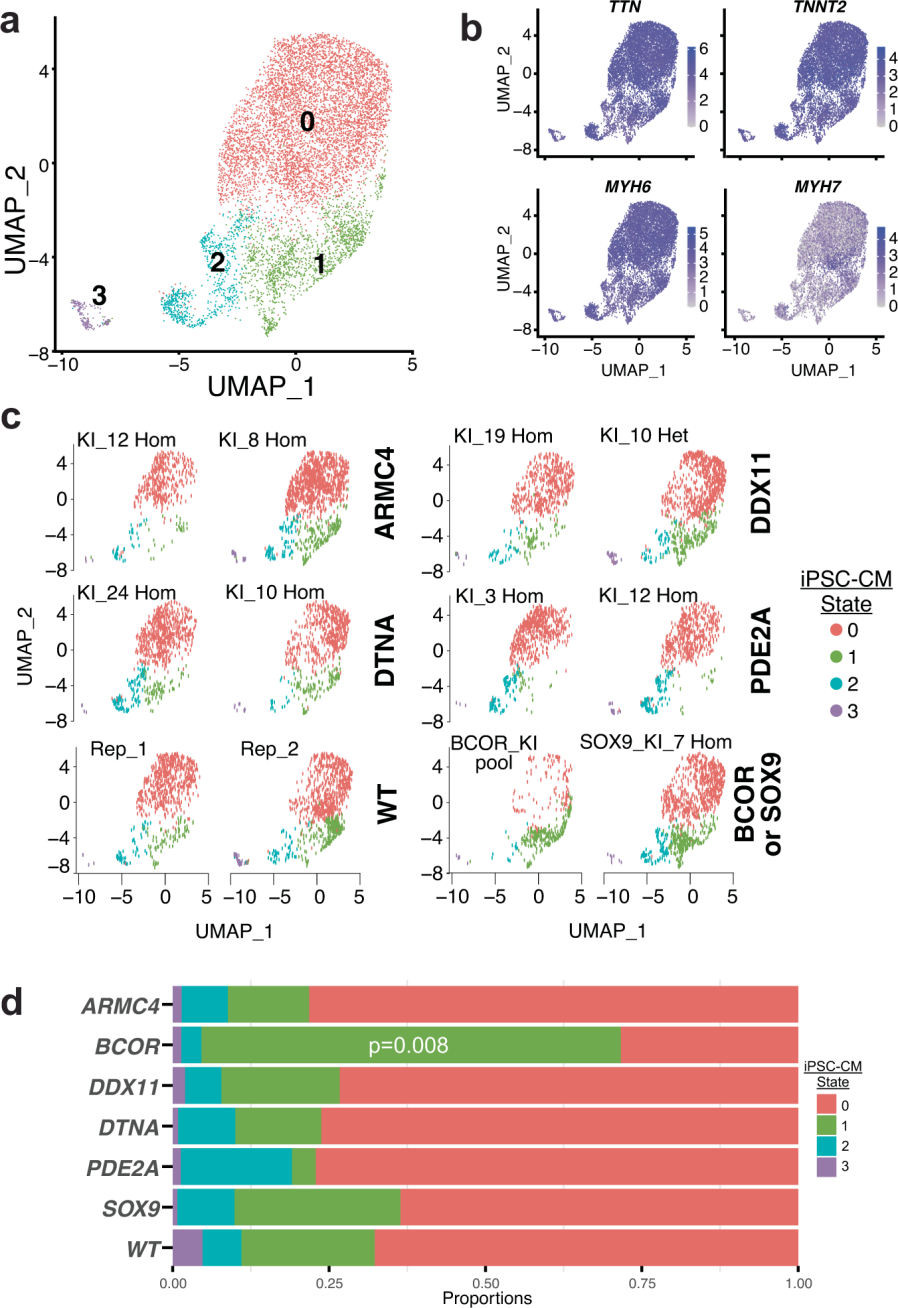
snRNA-seq characterization of the impact of five ncDNVs that did not alter MPRA activity in iPSC-CMs. Five ncDNVs that did not affect MPRA activity (MPRA-NC) and were knocked into WTC-11 iPSCs. **a**,**b**. Two independent knockin clones of ARMC4, DDX11, DTNA or PDE2A ncDNV, a SOX9 ncDNV knockin clone, a BCOR ncDNV knockin pool (positive control) and WTC-11 (two independent replicates) were differentiated into iPSC-CMs. On day 10, nuclei were analyzed by multiplexed snRNA-seq. Clustering identified 4 cell states (**a**) that express iPSC-CM markers (**b**). **c**. The distribution of iPSC-CMs among the 4 cell states was reproducible in biological replicate samples. **d**. Analysis of iPSC-CM state distribution by genotype. BCOR significantly expanded cluster 1 compared to WT (ANOVA with Dunnett’s test versus WT for each iPSC-CM state). The ncDNVs that did not affect MPRA activity had no significant effect on iPSC-CM state distribution.

In a control experiment, we introduced five ncDNVs that met the same selection criteria and that did not impact enhancer activity in the CHD lentiMPRA (Supplementary Table 4). Using the same multiplexed snRNA-seq approach, these clonal lines were differentiated into iPSC-CMs and their differentiation to iPSC-CMs was compared to wild-type iPS cells and a *BCOR* polyclonal ncDNV knock-in pool. This experiment identified four iPSC-CM cell states (Extended Data Fig. 8a,b). The *BCOR* ncDNV knock-in again altered the distribution of cell states compared to wild-type cells by significantly expanding cluster one. By contrast, the five ncDNVs that had no effect in the CHD lentiMPRA did not (Extended Data Fig. 8c,d).

Together, these data demonstrate that a subset of the functional ncDNVs identified by lentiMPRA regulate the expression of adjacent CHD genes when introduced into their native genomic context. Moreover, these ncDNVs have a substantial impact on iPS cell differentiation to cardiomyocytes, suggesting that they could affect heart development and contribute to CHD.

### Prediction of causal CHD DNVs using EpiCard

**Fig. 5 |:**
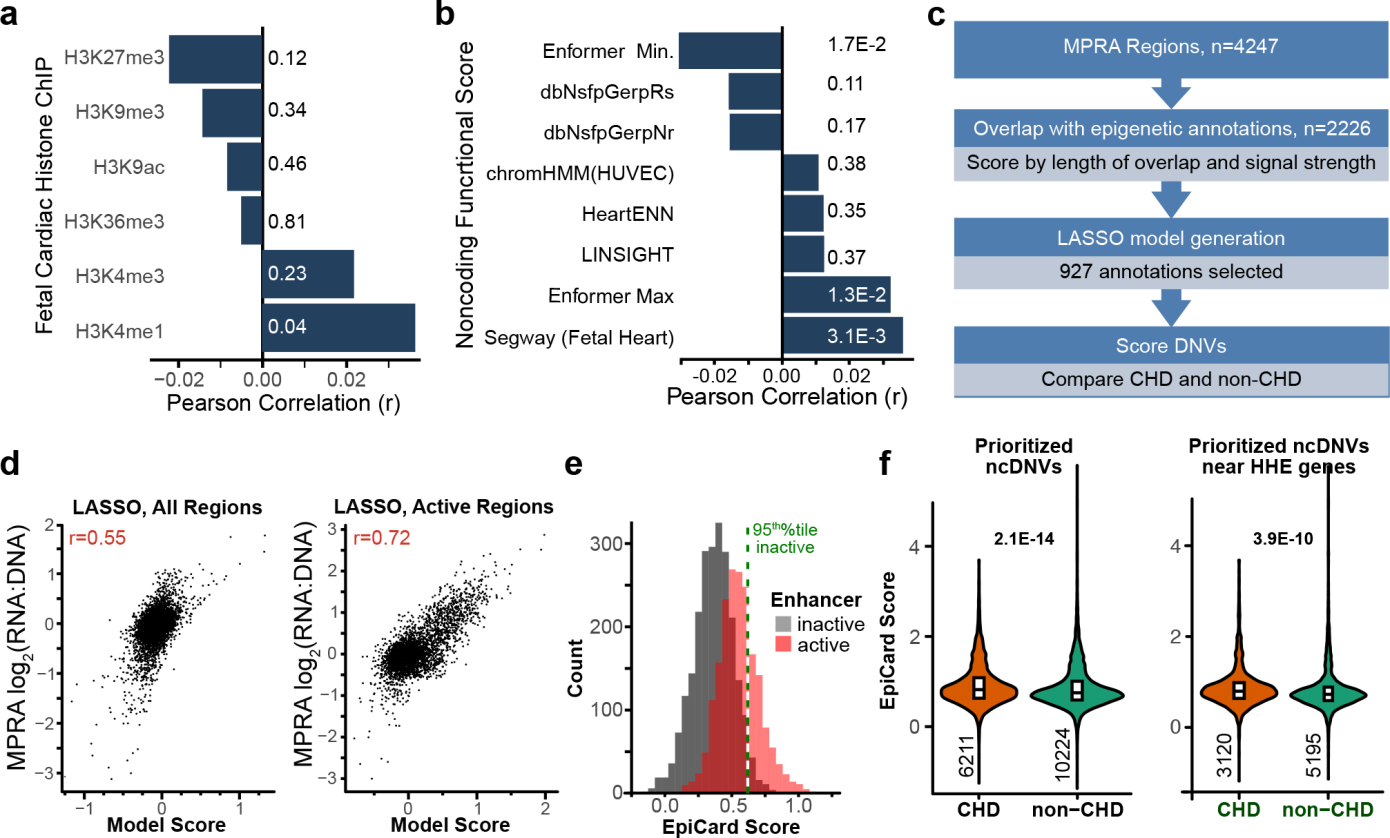
Development of ‘EpiCard’ noncoding functional score based on lentiMPRA enhancer activity measurements. **a**,**b**, Univariate correlations of MPRA activity with genomic annotations. Each bar is labeled with nominal Pearson *P* values. We did not detect a significant correlation with fetal cardiac histone marks (**a**). The maximum Enformer score and Segway noncoding functional score trained on fetal heart data were weakly correlated, whereas the minimum Enformer score was weakly anticorrelated (**b**). **c**, The EpiCard score used activity data from reference MPRA regions to train a model using epigenetic annotations The EpiCard score was then applied to ncDNVs, and values were compared between a CHD and non-CHD cohort. **d**, LASSO regression models trained on all data (left) or only the active MPRA regions (right) generated modest correlations. **e**, A binary model generated EpiCard scores that separated active and inactive regions. Green dotted line indicates the 95th percentile cutoff for inactive enhancers; active MPRA regions above that region were enriched (OR = 11.1; Fisher’s test, *P* < 2.2 × 10^−16^). **f**, EpiCard scores of ncDNVs identified in 1,062 independent CHD trios. Left, all ncDNVs meeting prioritization criteria (Fig. 3a). Right, subset of prioritized ncDNVs near HHE genes. EpiCard scores were significantly higher in CHD participants compared to non-CHD participants (two-sided *t*-test). Numbers by violins indicate number of ncDNVs in each group. Center and box indicate the median, 25th and 75th percentiles, respectively.

We next tested the hypothesis that MPRA results can be used to improve the prioritization of CHD ncDNVs. As each person has ~74 ncDNVs^6,7^, computational approaches are needed to identify ncDNVs that contribute to disease risk. First, we assessed the overlap of active MPRA regions with regions observed to interact with promoters in cardiomyocytes^33^. The overlap with active regions was greater than with inactive regions (443 out of 1,594 versus 665 out of 2,078 of 4,016 MPRA regions not in promoters from that dataset, odds ratio (OR) = 1.2, *P* = 9.8 × 10^−3^). However, only 27% of active regions were identified using this approach. We next assessed the association of MPRA activity with individual histone marks. The activities of 4,247 REF MPRA regions did not correlate well with histone mark annotations from the human fetal heart (Fig. 5a and Supplementary Table 6). Moreover, existing computational methods designed to estimate the regulatory potential of noncoding sequences poorly predicted MPRA activity—ChromHMM^10^, LINSIGHT^9^ and GERP^12^ scores were not correlated with MPRA activity; Segway^11^, trained on fetal heart annotations, was only weakly correlated (*r* = 0.04, *P* = 3.1 × 10^−3^; Fig. 5b); and Enformer^34^ minimum and maximum scores were nominally directionally correlated with MPRA activity (*r* = −0.04, *P* = 1.7 × 10^−2^ and *r* = 0.04, *P* = 1.3 × 10^−2^, respectively; Fig. 5b).

**Extended Data Fig. 9 |:**
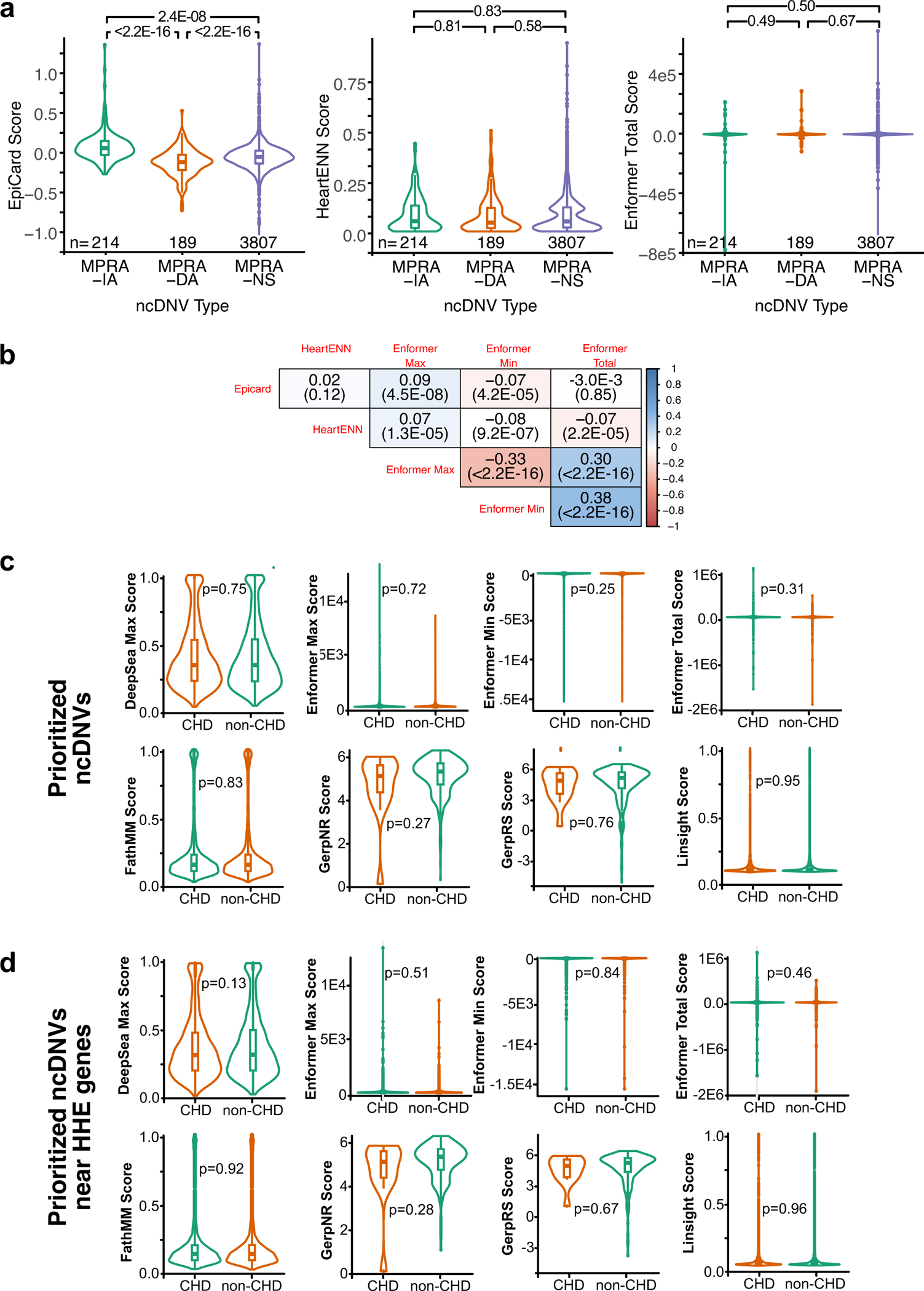
Characterization of EpiCard scores. **a**. Comparison of EpiCard, HeartENN and Enformer scores by MPRA region activity. Two-sided t-test. **b**. Correlation between EpiCard, HeartENN and Enformer scores expressed as Pearson coefficient (p-value) across 3745 ncDNVs with scores available. **c**,**d**. Comparison of functional scores for ncDNVs in an independent CHD cohort and non-CHD cohort, compared by 2-sided t-test with nominal p-values reported. **c.** All ncDNVs meeting prioritization criteria (see Fig. 3a). Right, subset of prioritized ncDNVs near HHE genes. ncDNVs (n = 6211 CHD and 10224 non-CHD). **d.** Subset of ncDNVs near HHE genes (n = 3120 CHD and 5195 non-CHD). DNVs.Center, box and whiskers indicate median, 25th and 75th percentiles and value closest to 25th percentile minus or 75th percentile plus 1.5 times the interquartile range.

We considered whether combinations of genomic annotations better modeled MPRA activity. We addressed this using a LASSO regression that included 2,226 epigenetic annotations, trained on MPRA activity (Fig. 5c). Using the entire dataset of active and inactive MPRA fragments, a model including 1,198 annotations had a Pearson correlation of 0.55 with MPRA activity (Fig. 5d, left). When subsetted to the 1,908 active MPRA fragments, a model including 954 annotations had a Pearson correlation of 0.72 with MPRA activity (Fig. 5d, right). When a binary LASSO was trained on active versus inactive MPRA regions, a 927-annotation model generated significantly higher scores for active regions (0.55 versus 0.36, *P* < 2.2 × 10^−16^; Fig. 5e). We denote this binary LASSO model as the EpiCard score. An EpiCard score above the 95th percentile value of inactive regions enriched for active MPRA regions (Fig. 5e, cutoff 0.50; 669 out of 1,908 active versus 116 out of 2,355, OR = 11.1, *P* < 2.2 × 10^−16^). EpiCard scores were higher in MPRA-DA regions compared to MPRA-IA or MPRA-NS regions (*P* < 2.2 × 10^−16^ for both; Supplementary Data 4 and Extended Data Fig. 9a, left) and lower for MPRA-IA regions compared to MPRA-NS regions (*P* = 2.4 × 10^−8^). This was expected because EpiCard was trained on REF sequences, which generally had activity in MPRA-DA and not MPRA-IA regions. There was no difference in EpiCard scores for MPRA variants in probands with different subtypes of CHD (conotruncal, right outflow tract obstruction, left outflow tract obstruction, or other). EpiCard scores did not differ significantly for MPRA regions from participants with and without reported neurodevelopmental delay.

We compared EpiCard to Enformer and HeartENN^6^, an algorithm previously developed to predict cardiac enhancers based on genomic and epigenomic data. HeartENN did not differ across MPRA-DA, MPRA-IA or MPRA-NS regions (mean = 0.084, 0.082 and 0.080, respectively; MPRA-DA versus MPRA-IA (*P* = 0.81), MPRA-DA versus MPRA-NS (*P* = 0.58), MPRA-IA versus MPRA-NS (*P* = 0.83); Extended Data Fig. 9a, middle). For each ncDNV, Enformer generates scores for multiple annotations, and therefore ncDNVs can be compared using the total, maximum or minimum Enformer values^34^. There was no overall difference in total, maximum or minimum Enformer scores between MPRA-IA, MPRA-DA and MPRA-NS regions (Extended Data Fig. 9a, right). For each MPRA region, EpiCard scores did not correlate with the HeartENN scores for the CHD ncDNV within the region (Extended Data Fig. 9b). EpiCard scores correlated weakly with maximum and minimum Enformer scores (Pearson *r* = 0.09, *P* = 4.5 × 10^−8^ and *r* = −0.07, *P* = 4.2 × 10^−5^, respectively; Extended Data Fig. 9b). However, EpiCard scores did not correlate with total Enformer or HeartENN scores (Extended Data Fig. 9b). These results indicate that the EpiCard scores reflect distinct parameters from those assessed by HeartENN and Enformer.

We evaluated EpiCard’s ability to prioritize ncDNVs in an independent set of 1,062 CHD trios and 1,610 non-CHD trios. The non-CHD trios comprised an unaffected sibling and parents from a study of autism spectrum disorder^7^. When including all ncDNVs, the average EpiCard score was higher among CHD participants (mean = 0.76 versus 0.71, *t*-test *P* = 2.1 × 10^−14^; Fig. 5f, left, and Supplementary Data 4). ncDNVs with EpiCard score above the 95th percentile value of the non-CHD DNVs were enriched in the CHD cohort (cutoff = 1.61; 380 out of 6,211 CHD ncDNVs versus 509 out of 10,224 non-CHD ncDNVs, OR = 1.2, *P* = 1.7 × 10^−3^) and present in 31% of the CHD cohort. After selecting only the highest scoring ncDNV per participant, there was also an enrichment for CHD participants with an EpiCard score >1.61 (326 out of 1,062 versus 435out of 1,610, OR = 1.2, *t*-test *P* = 0.04). Likewise, EpiCard scores for ncDNVs near HHE genes were higher in CHD participants (mean = 0.68 in CHD participants versus 0.62 in non-CHD participants, *P* = 3.9 × 10^−10^; Fig. 5f, right, and Supplementary Data 4). Previously reported variant prioritization scoring methods (DeepSea^35^, FathMM^36^, GERP^12^, LINSIGHT^9^ and Enformer) did not detect a significant difference between CHD and non-CHD cohorts at all ncDNVs or ncDNVs near HHE genes (Extended Data Fig. 9c,d). These results suggest that the EpiCard score will be useful in prioritizing CHD ncDNVs for burden analysis and functional testing.

## Discussion

Genome-wide association studies, WGS and pedigree studies indicate an important role of noncoding variants in modifying and causing human disease. Identifying and mechanistically studying these variants remain challenging owing to the complexities of prioritization and functional analysis. Our prior WGS study of CHD trios identified an increased burden of noncoding variants in CHD probands^6^, but we functionally interrogated only a small number of individual ncDNVs using traditional transfection of episomal luciferase reporters. Here we developed a robust high-throughput platform that functionally measures the impact of thousands of candidate ncDNVs on CRE activity. This platform enabled us to identify 403 CHD ncDNVs that impacted cardiac CRE activity and should enable systematic evaluation of ncDNVs in other conditions.

We found that ncDNVs in CHD probands had a similar likelihood of reducing the activity of REF enhancers (MPRA-DA) and conferring new enhancer activity to previously inactive sequences (MPRA-IA). This result suggests that ncDNVs may contribute to disease by inducing inappropriate enhancer activation, either by enabling ectopic transcription factor binding (for example, new SRF motif at *ADAMTS6* CRE) or by blocking repressor binding (for example, loss of HIC1/HIC2 motif adjacent to *GALNT6*). Prior ncDNV prioritization efforts have focused on identifying DNVs within active enhancers. Our results suggest that many impactful ncDNVs establish active enhancers that are not usually present. This class of ncDNVs would not be prioritized by strategies focused on enhancer prediction from reference genomes and epigenomes.

Efforts to understand the functional significance of noncoding variants require the development of robust approaches to predict CRE activities. Currently, the development of these tools is hamstrung by the scarcity of training data, which is largely attributable to the immense resource demands of transient transgenesis, the gold standard method of evaluating enhancer activity. The lentiMPRA platform enabled the quantitative measurement of enhancer activity of thousands of regions. Using this large dataset to train a classifier, EpiCard, we prioritized a subset of ncDNVs among all variants identified in CHD probands. Continued use of lentiMPRA in iPSC-CMs and other relevant cell types will expand the training dataset and may enable prediction of the candidate cell type affected by a noncoding variant.

Numerous cell types participate in heart development and each cell type changes dynamically during this process. An important limitation of our study is that it focused only on the cardiomyocyte lineage. The application of lentiMPRA to cardiac progenitors and other iPSC-derived lineages would likely uncover more functional ncDNVs that may contribute to CHD pathogenesis.

In summary, the combination of iPS cell differentiation and lentiMPRA enables the identification of ‘functional’ ncDNVs that likely contribute to CHD pathogenesis. We expect that this approach will be widely applicable to the analysis of noncoding variants in other conditions.

## Online content

Any methods, additional references, Nature Portfolio reporting summaries, source data, extended data, supplementary information, acknowledgements, peer review information; details of author contributions and competing interests; and statements of data and code availability are available at https://doi.org/10.1038/s41588-024-01669-y.

## Methods

### Institutional approvals

This study was performed in compliance with relevant ethical guidelines. Human study protocols were approved by Institutional Review Boards of Boston Children’s Hospital, Brigham and Women’s Hospital, Children’s Hospital of Los Angeles, Children’s Hospital of Philadelphia, Columbia University Medical Center, Great Ormond Street Hospital, Icahn School of Medicine at Mount Sinai, Rochester School of Medicine and Dentistry, Steven and Alexandra Cohen Children’s Medical Center of New York and Yale School of Medicine. Recombinant DNA, cells and viruses were used under protocols approved by the Boston Children’s Hospital Biosafety Committee.

### Human iPSC-CM differentiation

The WTC-11 hiPS cell line (Coriell Institute, GM25256) and its derivatives were cultured on Geltrex-precoated plates in mTeSR1 medium (STEMCELL Technologies, 85850). Generally, iPS cells were dissociated using Versene solution (Gibico, 15040066) and seeded into 12-well plates for the induction of iPSC-CM differentiation according to well-established protocols with some modifications^15,40^. In brief, 2 days after seeding into 12-well plates and when ~90% confluent, iPS cells were washed with PBS and treated with RPMI medium supplemented with B27 supplement (-insulin; Life Technologies, A1895601) and 7 μM CHIR99021 (STEMCELL Technologies, 72054). Forty-eight hours after CHIR99021 treatment, the medium was changed with the fresh basal medium of RPMI/B27. Twenty-four hours later, cells were treated with RPMI/B27 medium supplemented with 5 μM IWP2 (Tocris Bioscience, 3533) and XAV939 (Sigma-Aldrich, X3004). Forty-eight hours later, the medium was changed with basal RPMI/B27 medium every other day. At differentiation day 10, cells were dissociated with Accutase (STEMCELL Technologies, 07920) and replated into Geltrex-precoated six-well plates. Lactate selection was performed between day 12 and day 14 as described in ref. 41. iPSC-CMs were more than 90% cTNT+ after lactate selection, as assessed by FACS using anticardiac troponin T-FITC clone REA400 (Miltenyi Biotec; 1:50 dilution).

### Candidate cardiac enhancers

Candidate cardiac enhancers (*n* = 2,891) were identified using open chromatin regions identified from previously reported iPSC-CM ATAC-seq data^6^. Candidate regions were centered on ATAC-seq peak summits that were (1) present in day 8 iPSC-CMs and day 17 iPSC-CMs, (2) absent in iPS cells, and (3) near genes highly expressed in the developing mouse heart^3^. ATAC-seq peaks were annotated by ChIPseeker^42^, and promoters, exons and chromosome X/Y were excluded. Negative control regions (*n* = 802) were chosen from a set of 943 ATAC-seq peaks that were present in iPS cells but absent from iPSC-CMs at day 4, 8 and 17 of differentiation, and near genes highly expressed in iPS cells. Additionally, negative control regions (*n* = 57) were selected from exons of genes highly expressed in iPS cells but not iPSC-CMs.

For the mutagenesis MPRA, the top 123 active enhancers from the initial MPRA were selected for tiling mutagenesis. Each region was divided into three overlapping regions, and each region was represented by a wild-type 171 bp region and the same region with tiled 10 bp mutations. Negative control regions (*n* = 858) were selected from the same set of 943 ATAC-seq negative control candidate regions as the initial MPRA (726 shared regions).

### Participants

CHD participants were recruited to the Congenital Heart Disease Network Study (CHD GENES—ClinicalTrials.gov identifier: NCT01196182) of the Pediatric Cardiac Genomics Consortium (PCGC) as previously described^43^. All participants or their parents provided written informed consent using protocols that were reviewed and approved by the institutional review boards of participating institutions. Anonymized data and materials are available to qualified researchers trained in human participants confidentiality protocols at the National Institutes of Health dbGaP resource (dbgap.ncbi.nlm.nih.gov). Because the preponderance of participants were of European ancestry, we were unable to analyze the impact of genetic ancestry on ncDNV distribution.

### Selection of CHD ncDNVs for MPRA

DNVs to assess via MPRA were selected from 750 CHD participants^6^ based on annotation as noncoding and one or more of these qualifications—(1) prioritization via HeartENN^6^, (2) location within a VISTA fetal cardiac enhancer^6,44^ where the closest gene to that enhancer had ≥3 ncDNVs from patients with CHD, or (3) location within 20 kb of the transcriptional start site of a prioritized CHD gene (high heart expressed gene^3^, candidate human CHD gene, mouse CHD gene^4^ or a gene with multiple damaging coding DNVs within the PCGC cohort^4^; Supplementary Data 5). The closest gene to each ncDNV was determined by linear proximity as defined previously^45^. In a few cases, coding variants within exons or canonical splice sites were included in the MPRA library design; these were excluded from downstream analyses. Negative control regions (*n* = 865) were selected from the same set of negative control candidate regions used in the initial MPRA; this included all 858 from the mutagenesis MPRA. Oligos related to the top 18 most active regions in the mutagenesis MPRA were also included as positive controls.

### Massively parallel reporter assay

Lentivirus-mediated MPRA was conducted as previously described with some modifications^14^. For the MPRA to assess the enhancer activity of 400 bp regions, we designed pairs of self-priming, 230 nt oligos to obtain a 400 bp genomic region flanked by PCR primer sites. In brief, each enhancer consisted of two 230 nt oligonucleotides with 20 bp 3′ overlap. The 5′ ends of the left and right oligonucleotides had 20 bp primer binding sites. After pooled oligo synthesis (Agilent; Supplementary Data 1), the oligonucleotides within the pool were annealed and amplified by self-priming touch-down PCR using 2X Phusion HS Flex Master Mix (NEG, M0536S) and the following PCR program: 15 cycles (95 °C for 30 s, 75 °C (−1 °C per cycle) for 30 s, 75 °C for 1 min) followed by ten cycles (95 °C for 30 s, 60 °C for 30 s and 75 °C for 1 min). The touch-down PCR products were purified and amplified with adaptor primers (pLS-mP_STARR-F/R) for 20 additional cycles (95 °C for 30 s, 60 °C for 30 s and 72 °C for 1 min) using Phusion HS Flex DNA Polymerase (NEB, M0535). Then the purified PCR products were cloned by Gibson assembly (NEB, E2621S) into EcoRI-digested pLS-mP vector^14^ (Addgene, 81225) in the 3′ UTR of EGFP, such that the enhancer sequence drove its transcription into RNA, where it acted as its own barcode. For 10-bp tiling-deletion-based mutagenesis MPRA (Supplementary Data 2) and CHD ncDNV MPRA (Supplementary Data 3), each assay region was contained on a 230 nt oligonucleotide, with a 171 nt genomic region, a cloning site and a unique 15 nt barcode. The set of regions was synthesized as an oligonucleotide pool (Agilent HiFi Oligo Library). To maintain barcode-enhancer fidelity, the oligo pool was amplified for 12 cycles using 2X Phusion HS Flex Master Mix. The PCR amplicon was cloned by Gibson assembly into pLS-mP. We then inserted a minimal promoter and GFP reporter into the oligo cloning site such that the enhancer was upstream of the minimal promoter and the barcode was positioned within the 3′ UTR of the GFP reporter, as previously described^14^. Oligonucleotide sequences used for cloning are provided in Supplementary Table 5.

### RT–qPCR

Total RNA was isolated using the TRIzol Reagent (Invitrogen, 15596026) and was reverse transcribed to cDNA using the PrimeScript RT reagent Kit with gDNA Eraser (Takara, RR047A). Real-time qPCR was performed using PowerUp SYBR Green Master Mix (Applied Biosystems, A25742) and specific primers (Supplementary Table 5) on a Bio-Rad CFX384 real-time PCR system. *RPL37A* expression was used as an internal control to normalize the relative expression level of each gene.

### Enhancer CRISPRi

Cardiac enhancer CRISPRi was performed according to a recent study^21^. In brief, sgRNAs targeting enhancers were designed using CHOPCHOP^46^ and cloned into lenti-sgRNA-MS2-Puro (Addgene, 85413). Oligonucleotide sequences are provided in Supplementary Table 5. iPSC-CMs were cotransfected with lentivirus of sgRNA-MS2-Puro, MCP-LSD1-Hygro (Addgene, 138457) and dCas9-KRAB-BSD (Addgene, 90332). Two days after transfection, antibiotic selection was performed to increase CRISPRi efficiency. Seven days later, transfected iPSC-CMs were collected for target gene analysis by RT–qPCR.

### EMSA

EMSA was performed using a Thermo Fisher Scientific EMSA Kit (E33075) following the manufacturer’s instructions. Briefly, EMSA probes were synthesized and annealed on a heating block at 95 °C for 5 min and gradually cooled to room temperature. EMSA reactions (15–20 μl) included 1× binding buffer (150 mM KCl, 0.1 mM dithiothreitol, 0.1 mM EDTA, 10 mM Tris, pH 7.4), 0.1–2.0 μg recombinant human proteins and 500–800 fmol annealed probes. Reactions were incubated at room temperature for 20 min and then size-separated on a 6% nondenaturing polyacrylamide gel. DNA-bound complexes were visualized by staining with SYBR Green and imaging using a Bio-Rad Gel Doc XR+ system. Recombinant human proteins used in this study included SMAD2 (Abcam, ab85329), SRF (OriGene, TP308596), TBX20 (OriGene, TP762422), HIC2 (OriGene, TP760963), SOX9 (OriGene, TP308944) and GATA4 (OriGene, TP310945). The percentage of probe shifted was calculated by quantifying the free and shifted probe intensities using Fiji and then calculating shifted/(free + shifted). EMSA probe sequences are listed in Supplementary Table 5.

### CRISPR–Cas9-mediated genome editing

To introduce CHD ncDNVs into iPS cells, we used WTC-11 cells in which dox-inducible Cas9 (ref. 47) is inserted into the AAVS1 locus (WTC-Cas9 iPS cell line). sgRNAs targeting regions near the ncDNVs of interest were designed using CHOPCHOP^46^ and transcribed in vitro using the EnGen sgRNA Synthesis Kit (NEB, E3322S). Oligonucleotide sequences are in Supplementary Table 5. WTC-Cas9 iPS cells were treated with 2 μg ml^−1^ doxycycline for 12 h to induce Cas9 expression and then dissociated into single cells using Accutase. Then 2 μl of 50 μM homology-directed repair (HDR) template (171 nt ssODNs) and 5 μg sgRNAs were introduced into the doxycycline-treated iPS cells by nucleofection (Amaxa). Two days after nucleofection, iPS cells were dissociated with Accutase and 3,000 single iPS cells were seeded into one 10-cm dish precoated with Geltrex (Life Technologies, A1413302). Seven days later, single iPS cell clones were picked into 24-well plates for further culture and genotyping.

### Flow cytometry

Human iPSC-CMs were dissociated into single cells with Accutase at 37 °C for 10–20 min. Next, they were washed with 1× PBS and fixed with BD Cytofix/Cytoperm Fixation/Permeabilization Solution Kit (BD, 554714) for 20 min at room temperature. Fixed cells were washed with wash buffer and incubated with cTNT or isotype IgG antibodies (1:50) at 4 °C for 45 min or overnight. Then cells were washed twice with 2 ml wash buffer, resuspended with 0.5 ml wash buffer and filtered through a cell strainer into test tubes (Falcon, 352235). To quantify the GFP intensity of each enhancer, iPSC-CMs transduced with lentivirus of cardiac enhancers were dissociated with Accutase and washed with 1× PBS twice, then filtered through a cell strainer into test tubes. FACS analysis was performed on a BD FACS LSRFortessa.

### Cardiac enhancer MPRA data analysis

Cutadapt 2.5 (ref. 48) was used to remove primer sequences within each read. MPRA pair-end reads were aligned to hg19 using Bowtie2 (v.2.3.4.3)^49^ (--end-to-end) with default parameters. Next, a custom Python script was used to determine DNA and RNA read counts for each enhancer. Read counts were then normalized to sequencing depth (FPM). Regions covered by ≥20 FPM in at least one DNA library were kept for downstream analysis because the retention of regions with lower coverage reduced the correlation between replicates. We computed enhancer activity scores as the log_2_-transformed ratio $\left(\left({RNA}_{fpm}+1\right)/\left({DNA}_{fpm}+1\right)\right)$, where a pseudocount of 1 was added to both RNA and DNA counts. Enhancer activity scores for replicates were averaged. To identify elements with detectable enhancer activity, raw read counts were processed using DEseq2 (v.1.32.2)^37^. RNA and DNA counts were treated as distinct experimental conditions within each replicate. Active enhancers were defined as having a significantly elevated ratio of RNA to DNA counts with an adjusted *P* value < 0.05 (ref. 19). Enriched motifs were identified using Homer (v.4.11.1)^38^ and a previously described nonredundant motif database^50^. Subsequently, enriched motifs were annotated with all transcription factors belonging to the motif family. Only transcription factors with fragments per kilobase of transcript per million mapped reads (FPKM) > 1 in day 17 iPSC-CMs are shown in Figs. 2f,g and 3f,g, while Supplementary Data 6 includes all enriched motifs regardless of expression level. The code used for MPRA analysis is provided

We calculated an enrichment score that represents the distance between the cumulative probability of a specific group having different enhancer activity compared to the entire library (Extended Data Fig. 8). Define an MPRA library L with N elements: $L=\left(L_{j}|j=1,\ldots ,n\right)$ and a subset of MPRA regions of interest, R, with n members, $n\leq n:R=\left(r_{k}|k=1,\ldots ,n\right)$. The enrichment score E at a given position i is:

$$E_{R}(L,i)=\frac{1}{n}\sum_{t=1}^{i}\;\Lambda_{\left(r_{t}\in R\right)}-\frac{i}{n}$$

where Λ is an indicator function for membership in the specified gene set. A positive enrichment score indicates enrichment compared to the entire library, and a negative score indicates depletion. The enrichment P value for R was calculated by randomly selecting 2,000 region sets, each with the same number of elements as R. The permutation P value was the proportion of random sets whose mean enrichment score was greater (enrichment score of R>0) or smaller (enrichment score of R<0) than the mean enrichment score of R. The enrichment P value was corrected for multiple testing by the Bonferroni method.

### CHD MPRA library design

The CHD MPRA library was designed using a custom Python script, MPRA_library_designer.py (https://github.com/pulab/CHD_DNVs/tree/main/MPRA-Enhancer/MPRA_library_designer-main). For each ncDNV, a REF–ALT pair of oligonucleotides was designed, with 171 bp of REF genomic sequence centered on the variant (Fig. 3a). Each oligonucleotide was synthesized with a unique 15 bp barcode. To analyze next-generation sequencing data for the library, Cutadapt (v.2.5)^48^ was used to remove primer sequences. A custom Python script-mapped sequence reads library variants using the barcode. The remaining steps were performed as described above for the 400 bp enhancer MPRA library. To identify ncDNVs that significantly changed CRE activity, we used a custom R script to calculate the log_2_-transformed fold change activity between the REF and ALT pairs. Significance values were determined using the paired *t*-test, adjusted by the Benjamini–Hochberg (BH) method^51^. Differentially active pairs had $\left|{log}_{2}(FC)\right|\geq 0.58$, adjusted *P* value < 0.05 and detectable activity in at least one sample.

### Enhancer tiling mutagenesis

The tiling mutagenesis library was designed using a custom Python script, MPRA_library_designer.py (https://github.com/pulab/CHD_DNVs/tree/main/MPRA-Enhancer/MPRA_library_designer-main). Each 400 bp enhancer was divided into three overlapping fragments, and each fragment was covered by 10 bp deletion tiles (Fig. 3a). Each oligo was assigned a unique 15 bp barcode. Next-generation sequencing data and differential activity analysis were performed as described for the CHD MPRA library. Only regions with a valid wlid-type fragment were kept for downstream analysis.

### Analysis of the effect of sequence variants on transcription factor motifs

Fimo 4.12.0 (ref. 39) was used to identify motifs in each oligo within a window centered on the variant (±8 bp for CHD ncDNVs and ±10 bp for the mutagenesis library). Motifs were obtained from a nonredundant motif database^50^. Scores reported for each motif match were divided by a negative log_10_-transformed *P* value. Only motifs with *P* value < 1 × 10^−3^ in at least one oligo were kept for downstream analysis. For analysis of the effect of a sequence variant on a transcription factor motif, we applied a threshold of abs (Motif_score[ref]-Motif_score[alt]) ≥2, which is at least 100-FC in motif *P* value. The motif scores of all reference and variant oligo pairs were combined across the categories MPRA-IA_LoM, MPRA-IA_GoM, MPRA-DA_LoM and MPRA-DA_GoM. REF–ALT pairs in which the variant significantly changed enhancer activity were compared to control pairs in which variants did not significantly change enhancer activity. For each motif m, we calculated an enhancer activity change odds ratio as follows:

**Table:** 

For a motif *m*	Enhancer activity changed		
Motif score changed	–	Yes	No
	Yes	*P_mc_*	1–*P_mc_*
	No	*P_mm_*	1 – *P_mm_*

*P_mc_*=percentage of MPRA-IA or MPRA-DA enhancers with changed motif *m* binding score.

*P_mn_*=percentage of MPRA-IA or MPRA-DA enhancers without motif m changes


$$OR=\frac{n\;\times \;P_{mc}\;\times \;\left(1\;-\;P_{mn}\right)}{P_{mn}\;\times \;\left(1\;-\;P_{mc}\right)}$$

where n is a signed coefficient to indicate that the motif acted as an activator or repressor: n=1, motif binding score increased in MPRA-IA enhancers or decreased in MPRA-DA enhancers; n=-1, motif binding score decreased in MPRA-IA or increased in MPRA-DA.

Supplementary Data 6 includes all transcription factors. In scatterplots of motifs in each ALT–REF pair, each point represents one nonredundant motif family. Points were labeled with transcription factor names that were filtered for FPKM > 1 on day 17 iPSC-CMs.

### RNA-seq analysis

RNA-seq mapping and quantitation were done using STAR (v.2.6.1)^52^ with flags --quantMode TranscriptomeSAM --outSAMstrandField intronMotif with --genomeDir pointing to a hg38 STAR index. The mapped reads were further analyzed by HTSeq-count (v.0.11.2)^53^ and annotated using a RefSeq database^54^. Reads count were normalized by DEseq2 (v.1.32.2)^37^. The expression levels for each transcript were quantified by FPKM. For genes with multiple isoforms, the FPKM values were summed across all isoforms.

### Multiplexed snRNA-seq

Nuclei were prepared from frozen cell pellets of individual iPS cell lines differentiated to iPSC-CMs for 10 d. For BCOR, CRISPR editing was performed as described above, and editing products were used for differentiation without the selection of clonal lines. After barcoding using CellPlex (10X Genomics) and previously described protocols^55^, snRNA-seq libraries were prepared using Chromium 3′ v3.1 dual index (10X Genomics). Sequencing data were mapped to the human reference genome (hg38) with CellRanger. Doublet score was assigned by Scrublet^56^, and nuclei with doublet scores below 0.3 were included in the analysis. Data were analyzed in R using Seurat 4.3.0 (ref. 57). Nuclei were filtered to include only those with RNA 500–15,000, RNA features 300–6,000 and <5% mitochondrial reads. Nuclei were clustered based on the expression of the 2,500 most variable features, after scaling for RNA counts and mitochondrial percentage. UMAP projections were generated using 35 dimensions and a resolution of 0.4 and 0.2 for the functional and nonfunctional ncDNVs, respectively. Cell cluster proportions were compared by one-way analysis of variance (ANOVA) followed by Dunnett’s multiple comparison test in R using speckle 1.0.0 (ref. 58). Differential gene expression was analyzed using Seurat FindMarkers function with log_2_(FC) cutoff at 0.25 and min.pct cutoff at 0.25. *P* values were adjusted for multiple testing by the Benjamini–Hochberg method. Genes with adjusted *P* values less than 0.05 and significant in both replicates were used for GO analysis and differential gene expression heatmaps. GO analysis was performed using the R package clusterProfiler 4.8.1 (ref. 59).

### Integrative analysis of epigenetic annotations with MPRA regions

Epigenetic annotations (*n* = 2,226) were obtained from ENCODE and Roadmap Epigenomics (www.encodeproject.org), DeepBind^60^, Cistrome (cistrome.org/), GWAS catalog (www.ebi.ac.uk/gwas) and individual publications (Supplementary Table 7). For 1,050 files in hg19, UCSC-liftOver^61^ was used to convert to hg38. The total length of unmapped intervals was 0.26% of the hg19 bed file interval lengths, with a median of 0.82% and interquartile range of 0.03–1.2%. Some datasets contained quantitative information such as peak height for ATAC-seq, while others were genomic locations only. Overlap between an MPRA region and each annotation was determined using bedtools^62^. Each MPRA region:annotation pair was assigned a score based on the length of overlap with an annotation (all annotations), and, for all annotations with quantitative traits, the average and total annotation value in the overlap with an annotation.

### Modeling of MPRA activity

A LASSO model with fivefold cross-validation was implemented using the R package glmnet 4.1–7 (ref. 63) to generate a model that predicted the RNA:DNA ratio from the REF MPRA assays. First, RNA:DNA ratio values were log-transformed to produce a normal distribution. Next, a LASSO model was fit to either the entire MPRA dataset or the subset of regions determined to be active by DESeq2 as detailed above. The final model was selected using the identified lambda divided by ten to reduce overfitting. Pearson correlation was calculated for the RNA:DNA ratio and LASSO score.

### EpiCard scores from independent CHD and non-CHD trios

EpiCard scores were calculated genome-wide for ncDNVs from an independent cohort of 2,673 probands and their parents, where 1,062 probands had CHD and 1,610 did not have CHD. First, 6,497 ncDNVs in CHD participants and 10,357 ncDNVs in non-CHD participants were selected based on the same principles as those assessed by MPRA (Supplementary Data 7), namely location within enhancers and/or neighboring CHD-associated genes. EpiCard scores were then calculated for the 200 bp region centered on the ncDNV using the weightings determined by the binary LASSO model trained on REF MPRA activity.

### Statistics and reproducibility

Experiments were performed using objective, quantitative assays. No statistical method was used to predetermine the sample size. No data were excluded from the analyses. The experiments were not randomized. The investigators were not blinded to allocation during experiments and outcome assessment.

Statistical analysis was performed in R, Prism and Excel. R analysis was supported by tidyverse (ver. 1.3.1). Specific statistical tests are indicated in each figure legend. Data distribution was assumed to be normal, but this was not formally tested.

## Extended Data

**Extended Data Fig. 10 |:**
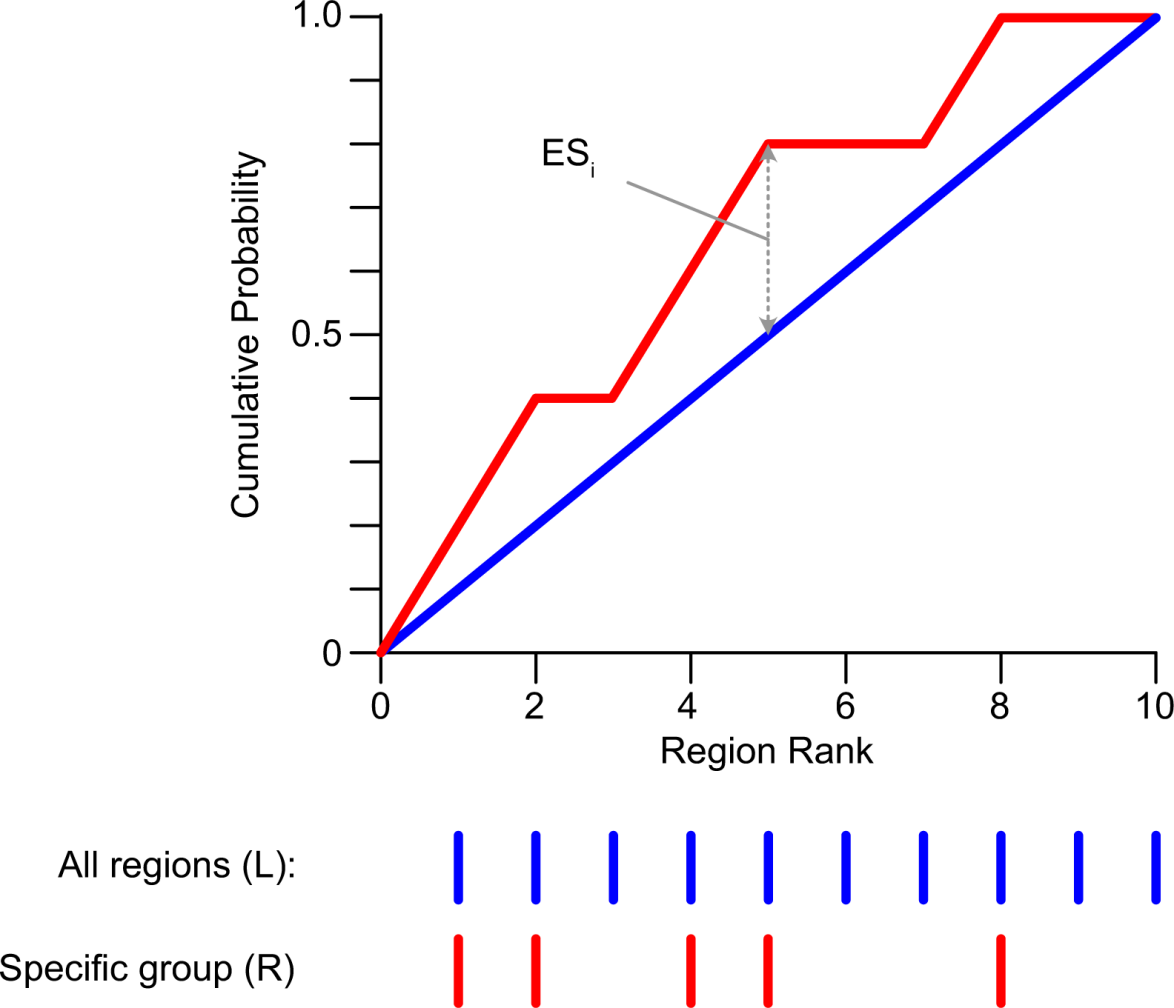
Schematic of enrichment score calculation. Given a ranked list L and a specific group of regions R that is a subset of L, the enrichment score at position i (ES_i_) is the difference between the cumulative probability of membership in R compared to L.

## Supplementary Material

Supplemental Data. 1

Supplemental Data.2

Supplemental Data.4

Supplemental Data.3

Supplemental Data.5

Supplemental Data.6

Supplemental Data.7

Supplemental Tables.1_7.

## Data Availability

RNA-seq and MPRA next-generation sequencing data associated with this study have been deposited to Gene Expression Omnibus (GSE208283 and GSE210376). WGS data were reported previously^6,7^ and are available through dbGaP (phs001138.v4.p2, phs001194.v3.p2 and phs001735.v2.p1). Source data are provided with this paper.

## References

[1] Van der Linde D Birth prevalence of congenital heart disease worldwide: a systematic review and meta-analysis. J. Am. Coll. Cardiol. 58, 2241–2247 (2011). 22078432 10.1016/j.jacc.2011.08.025

[2] Zaidi S De novo mutations in histone-modifying genes in congenital heart disease. Nature 498, 220–223 (2013). 23665959 10.1038/nature12141 PMC3706629

[3] Homsy J De novo mutations in congenital heart disease with neurodevelopmental and other congenital anomalies. Science 350, 1262–1266 (2015). 26785492 10.1126/science.aac9396 PMC4890146

[4] Jin SC Contribution of rare inherited and de novo variants in 2,871 congenital heart disease probands. Nat. Genet. 49, 1593–1601 (2017). 28991257 10.1038/ng.3970 PMC5675000

[5] ENCODE Project Consortium An integrated encyclopedia of DNA elements in the human genome. Nature 489, 57–74 (2012). 22955616 10.1038/nature11247 PMC3439153

[6] Richter F Genomic analyses implicate noncoding de novo variants in congenital heart disease. Nat. Genet. 52, 769–777 (2020).32601476

[7] Morton SU Genome-wide de novo variants in congenital heart disease are not associated with maternal diabetes or obesity. Circ. Genom. Precis. Med. 15, e003500 (2022). 35130025 10.1161/CIRCGEN.121.003500 PMC9295870

[8] Blow MJ ChIP–seq identification of weakly conserved heart enhancers. Nat. Genet. 42, 806–810 (2010). 20729851 10.1038/ng.650 PMC3138496

[9] Huang Y-F, Gulko B & Siepel A Fast, scalable prediction of deleterious noncoding variants from functional and population genomic data. Nat. Genet. 49, 618–624 (2017). 28288115 10.1038/ng.3810 PMC5395419

[10] Ernst J & Kellis M Chromatin-state discovery and genome annotation with ChromHMM. Nat. Protoc. 12, 2478–2492 (2017). 29120462 10.1038/nprot.2017.124 PMC5945550

[11] Hoffman MM, Buske OJ, Wang J, Weng Z & Bilmes JA Unsupervised pattern discovery in human chromatin structure through genomic segmentation. Nat. Methods 9, 473–476 (2012). 22426492 10.1038/nmeth.1937 PMC3340533

[12] Cooper GM Distribution and intensity of constraint in mammalian genomic sequence. Genome Res. 15, 901–913 (2005). 15965027 10.1101/gr.3577405 PMC1172034

[13] Inoue F & Ahituv N Decoding enhancers using massively parallel reporter assays. Genomics 106, 159–164 (2015). 26072433 10.1016/j.ygeno.2015.06.005 PMC4540663

[14] Inoue F A systematic comparison reveals substantial differences in chromosomal versus episomal encoding of enhancer activity. Genome Res. 27, 38–52 (2017). 27831498 10.1101/gr.212092.116 PMC5204343

[15] Lian X Directed cardiomyocyte differentiation from human pluripotent stem cells by modulating Wnt/β-catenin signaling under fully defined conditions. Nat. Protoc. 8, 162–175 (2013). 23257984 10.1038/nprot.2012.150 PMC3612968

[16] Barakat TS Functional dissection of the enhancer repertoire in human embryonic stem cells. Cell Stem Cell 23, 276–288 (2018). 30033119 10.1016/j.stem.2018.06.014 PMC6084406

[17] Visel A, Minovitsky S, Dubchak I & Pennacchio LA VISTA enhancer browser—a database of tissue-specific human enhancers. Nucleic Acids Res. 35, D88–D92 (2007). 17130149 10.1093/nar/gkl822 PMC1716724

[18] Arnold CD Genome-wide quantitative enhancer activity maps identified by STARR-seq. Science 339, 1074–1077 (2013). 23328393 10.1126/science.1232542

[19] Tewhey R Direct identification of hundreds of expression-modulating variants using a multiplexed reporter assay. Cell 165, 1519–1529 (2016). 27259153 10.1016/j.cell.2016.04.027 PMC4957403

[20] Klein JC A systematic evaluation of the design and context dependencies of massively parallel reporter assays. Nat. Methods 17, 1083–1091 (2020). 33046894 10.1038/s41592-020-0965-y PMC7727316

[21] Li K Interrogation of enhancer function by enhancer-targeting CRISPR epigenetic editing. Nat. Commun. 11, 485 (2020). 31980609 10.1038/s41467-020-14362-5 PMC6981169

[22] Hilton EN Left-sided embryonic expression of the BCL-6 corepressor, BCOR, is required for vertebrate laterality determination. Hum. Mol. Genet. 16, 1773–1782 (2007). 17517692 10.1093/hmg/ddm125

[23] Hamline MY OFCD syndrome and extraembryonic defects are revealed by conditional mutation of the polycomb-group repressive complex 1.1 (PRC1.1) gene BCOR. Dev. Biol. 468, 110–132 (2020). 32692983 10.1016/j.ydbio.2020.06.013 PMC9583620

[24] Wang D Activation of cardiac gene expression by myocardin, a transcriptional cofactor for serum response factor. Cell 105, 851–862 (2001). 11439182 10.1016/s0092-8674(01)00404-4

[25] Huang J Myocardin regulates BMP10 expression and is required for heart development. J. Clin. Invest. 122, 3678–3691 (2012). 22996691 10.1172/JCI63635 PMC3461917

[26] Houweling AC. et al. Loss-of-function variants in myocardin cause congenital megabladder in humans and mice. J. Clin. Invest. 129, 5374–5380 (2019). 31513549 10.1172/JCI128545 PMC6877301

[27] Santamaria S & de Groot R ADAMTS proteases in cardiovascular physiology and disease. Open Biol. 10, 200333 (2020). 33352066 10.1098/rsob.200333 PMC7776578

[28] Prins BP Exome-chip meta-analysis identifies novel loci associated with cardiac conduction, including ADAMTS6. Genome Biol. 19, 87 (2018). 30012220 10.1186/s13059-018-1457-6 PMC6048820

[29] Tian E Galnt1 is required for normal heart valve development and cardiac function. PLoS ONE 10, e0115861 (2015). 25615642 10.1371/journal.pone.0115861 PMC4304789

[30] Dykes IM HIC2 is a novel dosage-dependent regulator of cardiac development located within the distal 22q11 deletion syndrome region. Circ. Res. 115, 23–31 (2014). 24748541 10.1161/CIRCRESAHA.115.303300

[31] Zhang Q Multiplexed single-nucleus RNA sequencing using lipid-oligo barcodes. Curr. Protoc. 2, e579 (2022). 36286606 10.1002/cpz1.579 PMC9614549

[32] Wang Z A non-canonical BCOR-PRC1.1 complex represses differentiation programs in human ESCs. Cell Stem Cell 22, 235–251 (2018). 29337181 10.1016/j.stem.2017.12.002 PMC5797497

[33] Montefiori LE A promoter interaction map for cardiovascular disease genetics. eLife 7, e35788 (2018). 29988018 10.7554/eLife.35788 PMC6053306

[34] Avsec Ž Effective gene expression prediction from sequence by integrating long-range interactions. Nat. Methods 18, 1196–1203 (2021). 34608324 10.1038/s41592-021-01252-x PMC8490152

[35] Zhou J & Troyanskaya OG Predicting effects of noncoding variants with deep learning-based sequence model. Nat. Methods 12, 931–934 (2015). 26301843 10.1038/nmeth.3547 PMC4768299

[36] Shihab HA An integrative approach to predicting the functional effects of non-coding and coding sequence variation. Bioinformatics 31, 1536–1543 (2015). 25583119 10.1093/bioinformatics/btv009 PMC4426838

[37] Love MI, Huber W & Anders S Moderated estimation of fold change and dispersion for RNA-seq data with DESeq2. Genome Biol. 15, 550 (2014). 25516281 10.1186/s13059-014-0550-8 PMC4302049

[38] Heinz S Simple combinations of lineage-determining transcription factors prime *cis*-regulatory elements required for macrophage and B cell identities. Mol. Cell 38, 576–589 (2010). 20513432 10.1016/j.molcel.2010.05.004 PMC2898526

[39] Grant CE, Bailey TL & Noble WS FIMO: scanning for occurrences of a given motif. Bioinformatics 27, 1017–1018 (2011). 21330290 10.1093/bioinformatics/btr064 PMC3065696

[40] Hamad S Generation of human induced pluripotent stem cell-derived cardiomyocytes in 2D monolayer and scalable 3D suspension bioreactor cultures with reduced batch-to-batch variations. Theranostics 9, 7222–7238 (2019). 31695764 10.7150/thno.32058 PMC6831300

[41] Tohyama S Distinct metabolic flow enables large-scale purification of mouse and human pluripotent stem cell-derived cardiomyocytes. Cell Stem Cell 12, 127–137 (2013). 23168164 10.1016/j.stem.2012.09.013

[42] Yu G, Wang L-G & He Q-Y ChIPseeker: an R/Bioconductor package for ChIP peak annotation, comparison and visualization. Bioinformatics 31, 2382–2383 (2015). 25765347 10.1093/bioinformatics/btv145

[43] Hoang TT The Congenital Heart Disease Genetic Network Study: cohort description. PLoS ONE 13, e0191319 (2018). 29351346 10.1371/journal.pone.0191319 PMC5774789

[44] Dickel DE Genome-wide compendium and functional assessment of in vivo heart enhancers. Nat. Commun. 7, 12923 (2016). 27703156 10.1038/ncomms12923 PMC5059478

[45] McLean CY GREAT improves functional interpretation of *cis*-regulatory regions. Nat. Biotechnol. 28, 495–501 (2010). 20436461 10.1038/nbt.1630 PMC4840234

[46] Labun K CHOPCHOP v3: expanding the CRISPR web toolbox beyond genome editing. Nucleic Acids Res. 47, W171–W174 (2019). 31106371 10.1093/nar/gkz365 PMC6602426

[47] Mandegar MA CRISPR interference efficiently induces specific and reversible gene silencing in human iPSCs. Cell Stem Cell 18, 541–553 (2016). 26971820 10.1016/j.stem.2016.01.022 PMC4830697

[48] Martin M Cutadapt removes adapter sequences from high-throughput sequencing reads. EMBnet J. 17, 10–12 (2011).

[49] Langmead B, Trapnell C, Pop M & Salzberg SL Ultrafast and memory-efficient alignment of short DNA sequences to the human genome. Genome Biol. 10, R25 (2009). 19261174 10.1186/gb-2009-10-3-r25 PMC2690996

[50] Vierstra J Global reference mapping of human transcription factor footprints. Nature 583, 729–736 (2020). 32728250 10.1038/s41586-020-2528-x PMC7410829

[51] Dubitzky W., Wolkenhauer O., Cho K-H. & Yokota H. (eds.). Encyclopedia of Systems Biology, pp. 78 (Springer, 2013).

[52] Dobin A STAR: ultrafast universal RNA-seq aligner. Bioinformatics 29, 15–21 (2013). 23104886 10.1093/bioinformatics/bts635 PMC3530905

[53] Anders S, Pyl PT & Huber W HTSeq—a Python framework to work with high-throughput sequencing data. Bioinformatics 31, 166–169 (2015). 25260700 10.1093/bioinformatics/btu638 PMC4287950

[54] O’Leary NA Reference sequence (RefSeq) database at NCBI: current status, taxonomic expansion, and functional annotation. Nucleic Acids Res. 44, D733–D745 (2016). 26553804 10.1093/nar/gkv1189 PMC4702849

[55] Nadelmann ER Isolation of nuclei from mammalian cells and tissues for single-nucleus molecular profiling. Curr. Protoc. 1, e132 (2021). 34043278 10.1002/cpz1.132 PMC8191490

[56] Wolock SL, Lopez R & Klein AM Scrublet: computational identification of cell doublets in single-cell transcriptomic data. Cell Syst. 8, 281–291 (2019). 30954476 10.1016/j.cels.2018.11.005 PMC6625319

[57] Hao Y Integrated analysis of multimodal single-cell data. Cell 184, 3573–3587 (2021). 34062119 10.1016/j.cell.2021.04.048 PMC8238499

[58] Phipson B propeller: testing for differences in cell type proportions in single cell data. Bioinformatics 38, 4720–4726 (2022). 36005887 10.1093/bioinformatics/btac582 PMC9563678

[59] Yu G, Wang L-G, Han Y & He Q-Y clusterProfiler: an R package for comparing biological themes among gene clusters. OMICS 16, 284–287 (2012). 22455463 10.1089/omi.2011.0118 PMC3339379

[60] Alipanahi B, Delong A, Weirauch MT & Frey BJ Predicting the sequence specificities of DNA- and RNA-binding proteins by deep learning. Nat. Biotechnol. 33, 831–838 (2015). 26213851 10.1038/nbt.3300

[61] Kent WJ The human genome browser at UCSC. Genome Res. 12, 996–1006 (2002). 12045153 10.1101/gr.229102 PMC186604

[62] Quinlan AR & Hall IM BEDTools: a flexible suite of utilities for comparing genomic features. Bioinformatics 26, 841–842 (2010). 20110278 10.1093/bioinformatics/btq033 PMC2832824

[63] Friedman J, Hastie T & Tibshirani R Regularization paths for generalized linear models via coordinate descent. J. Stat. Softw. 33, 1–22 (2010). 20808728 PMC2929880

[64] Zhang X, Morton SU, Seidman JG, Seidman CS & Pu WT Analysis code used to analyze ncDNVs in CHD. Zenodo https://zenodo.org/records/10294614 (2024).

